# Thermal Utilization on Chip

**DOI:** 10.1038/s41377-026-02326-1

**Published:** 2026-06-02

**Authors:** Yaohao Zhang, Bo Lai, Fei Yu, Xuesong Li, Yue Yang, Wei Lü, Ke Jiang, Xiaojuan Sun, Dabing Li

**Affiliations:** 1 State Key Laboratory of Luminescence Science and Technology, Changchun Institute of Optics, Fine Mechanics and Physics, Chinese Academy of Sciences, Changchun, China; 2 Key Laboratory of Advanced Structural Materials, Ministry of Education and School of Materials Science and Engineering and Advanced Institute of Materials Science, Changchun University of Technology, Changchun, China; 3 Center of Materials Science and Optoelectronics Engineering, University of Chinese Academy of Sciences, Beijing, China

**Keywords:** Electronics, photonics and device physics, Lasers, LEDs and light sources

## Abstract

The integration and miniaturization of chips lead to significant power consumption and heat accumulation. Typically, the energy consumption of cooling systems accounts for morn than 50% of the input energy. Current thermal management technologies do not offer solutions for on-chip thermal energy loss. Herein, we propose an on-chip integrated thermal recovery system, which can simultaneously achieve efficient heat dissipation. Present system on chips is based on hydrovoltaic generator technology, consisting of electrodes and gel. With the deep ultraviolet LED (236 nm) chip suffering from severe heat accumulation as a prototype, upon integration with the thermal recovery system, not only maintain the chip temperature below 40 °C, but also converts waste heat into stored electrical energy, resulting in a 610.70% improvement in overall energy utilization efficiency. To demonstrate its general applicability in commercial CPU systems, we used the commercial Intel G3220 chip and as an example, by incorporating four HEG units, the temperature was reduced from 93 °C to below 60 °C, effectively enhancing computational performance and extending the chip’s lifespan.

## Introduction

With the continuous emergence of new fields such as 5 G, artificial intelligence, and deep space exploration, the demand for chip computing power is steadily increasing, leading to a rise in chip integration density^1–4^. Consequently, as integration density continues to increase, the heat flux density generated by chips also escalates, making on-chip thermal management increasingly challenging^5,6^. In practical applications, chips commonly utilize cooling methods such as air cooling^7,8^, water cooling^9,10^, heat pipes^11,12^, and microchannels^13,14^ to enhance cooling efficiency by improving the thermal conductivity and specific surface area of materials, thereby addressing the growing thermal management demands^15,16^. Additionally, radiative cooling methods have garnered attention, as they convert heat into infrared wavelengths and radiate it into space^17,18^. However, these methods either fail to meet high cooling requirements or necessitate additional energy for heat transfer. Even in large data centers, cooling energy consumption can account for up to 50% of total energy consumption, resulting in significant energy loss.

A strategy that can realize heat dissipatation and utilization simultaneously is highly desired. For waste thermal recovery, in large-scale industrial applications, however, efficient heat utilization facilities are in place^19,20^. For example, in the steel smelting industry, water pipes are deployed on the furnace walls of blast furnaces. The waste heat generated by the furnace turns the water into steam, which then drives a steam generator to produce electrical energy^21^. However, the method of converting thermal energy into mechanical energy and then into electrical energy requires significant space^22–24^, making it impossible to deploy similar devices within the limited space of a chip.

A power generation technology based on the relative movement of water molecules and materials is expected to fill this gap^25–27^. By collecting the current generated from charge transfer caused by water molecules passing over the surface of the material, or from the ionic directional movement resulting from the absorption of water molecules from the air, this technology has developed over the years to gradually provide power for small electronic devices^28–31^. Both evaporation-driven power generation and moisture-driven power generation are directly controlled by surrounding heat^32–34^. Under heat-driven conditions, water molecules undergo random thermal motion, generating electrical signals through adsorption and evaporation^35–37^. Furthermore, water molecules are excellent thermal carriers, with the highest specific heat capacity among common substances, reaching 4.18 J (g K)^-1^. Therefore, hydroelectric generation technology holds immense potential in waste heat management and recovery. Through hydroelectric electricity generators (HEG), it is expected to achieve both chip cooling and energy conversion.

Herein, we have designed an on-chip energy harvesting based on thermally conductive aerogel HEG. By constructing a composite aerogel from carboxymethyl cellulose and sodium hyaluronate, it absorbs moisture from the air, creating an internal ion gradient. As a result, under a 25 °C, 100% humidity environment, it generates an open-circuit voltage of 0.78 V and a current density of 0.61 mA cm^-2^, with a peak power density reaching 93.14 μW cm^-2^. To test the heat dissipation capability of HEG, a DUV LED with a center wavelength of 236 nm was fabricated. Under an input current of 100 mA, the luminous power reached 1.48 mW. The optimal power of the HEG also increases to 162.08 μW cm^-2^, and the overall energy utilization efficiency improves by 610.70%. The electrical energy generated with the assistance of Na-ion batteries can indirectly drive DUV LEDs, achieving energy cycling. When applied to the commercial Intel G3220 chip, the operating temperature was reduced from 93 °C to below 60 °C, the maximum processing rate increased by 16.6%, and thermal energy was successfully converted into electrical energy. Furthermore, the applications of radio frequency amplifier (RFA), high-power LEDs, and motors also demonstrate the universal applicability of HEG in the thermal utilization of electronic devices.

## On-chip thermal utilization structure and characterization

**Fig. 1:**
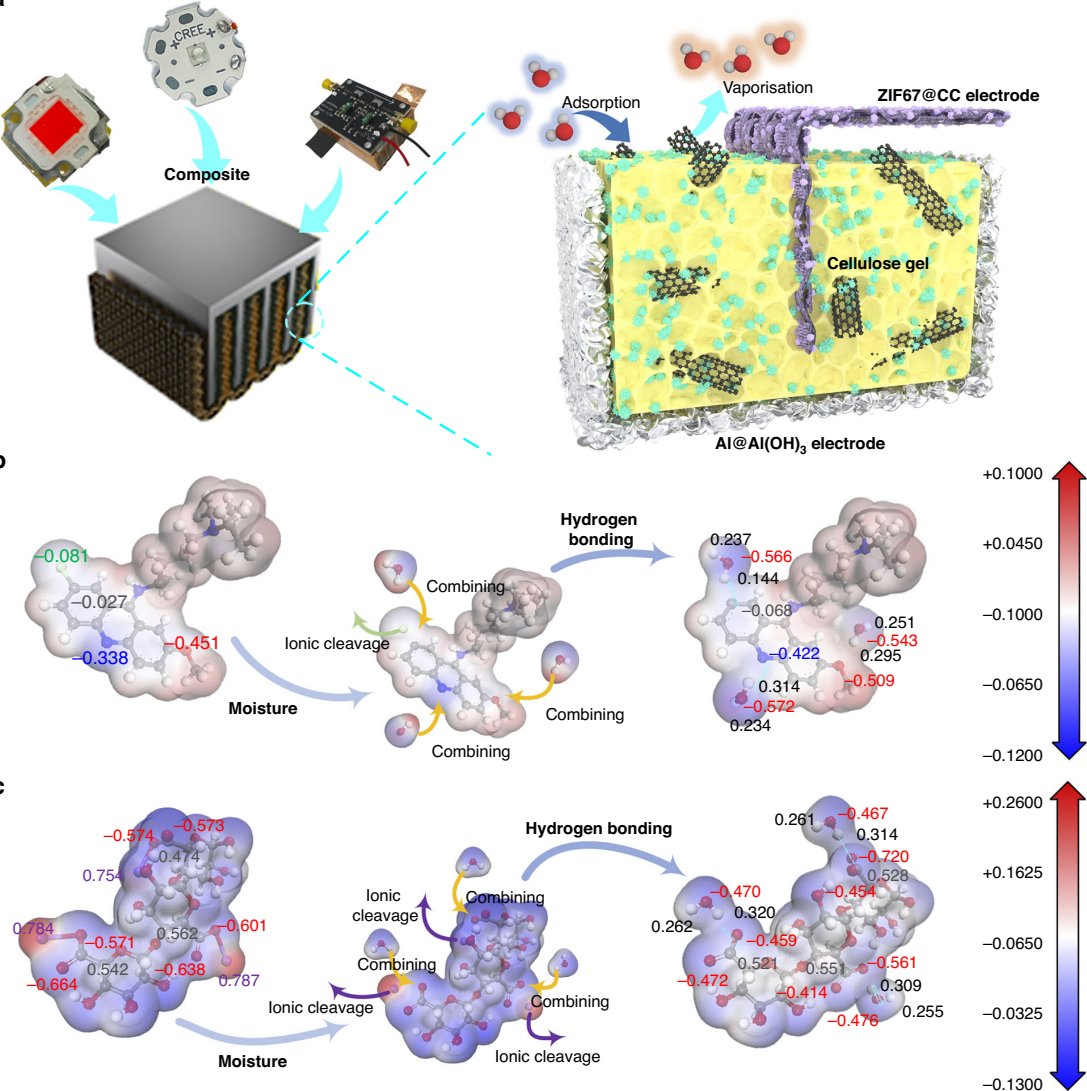
Device structure and gel molecular charge map. **a** Schematic diagram of the LED(236 nm)@HEG. Changes in the surface charge density before and after the binding of **b** CMC-C and **c** SA molecules with water molecules

The proposed on-chip thermal utilization system (Fig. 1a) design includes an Al electrode, a thermally conductive composite gel, and a carbon cloth (CC) composite electrode. As described in Figure S1 (SI), the Al electrode undergoes superhydrophilic treatment with thermal alkali to form Al(OH)_3_ nanosheets on its surface (Figure S2, SI), which enhances the electrode’s specific surface area and heat exchange area. At the same time, the fin-shaped Al electrodes play a crucial role in the stability of the HEG device, ensuring that the fragile aerogel does not crack or fail during operation. The device remains stable under a pressure of 4 MPa (Figure S3, SI). As for the CC electrode, treated with ZIF-67 composite (Figure S4 and S5, SI), effectively improves the specific surface area, and the increase in oxygen-containing groups also facilitates hydrogen bonding with water molecules (Figure S6, SI). More importantly, ZIF-67 effectively enhances the surface charge of the CC electrode (Figure S7, SI), helping to increase the open-circuit voltage of the HEG and enhance the chip’s thermal recovery efficiency.

The thermally conductive composite aerogel has calcium carboxymethyl cellulose (CMC-C) as the core material, with multi-walled carbon nanotubes (MWCNT) added to improve thermal conductivity. Components such as sodium hyaluronate (SH) and sodium alginate (SA) are incorporated into the gel, which, as evidenced by Fourier-transform infrared spectroscopy (FTIR) and X-ray photoelectron spectroscopy (XPS) tests (Figure S8 and S9, SI), show a significant increase in the number of oxygen-containing groups, effectively enhancing the aerogel’s water retention capacity. As a result, only 36.1 mg of the composite aerogel can absorb 376.5 mg of water (Figure S10, SI). When the chip is idle, the HEG spontaneously absorbs water vapor from the air and stores it. The ions in the gel hydrolyze, and under the ion gradient, they move in a directed manner, generating current. When the chip is operating, the stored water absorbs heat and evaporates, taking away heat from the chip to regulate its temperature. At the same time, as the temperature increases, ion hydrolysis intensifies, increasing the number of charge carriers, which effectively enhances the electrical signal strength.

The aerogel filling between the electrodes is also a key factor affecting the HEG’s power generation performance. When water molecules contact with aerogel molecules, internal electrons undergo significant migration. Figure 1b, illustrates the surface charge changes caused by the adsorption of three water molecules onto CMC-C. CMC-C serving as the aerogel matrix, contains three nitrogen (N) atoms, one oxygen (O) atom, and one chlorine (Cl) atom per molecule. When CMC-C is in a dry state, Cl atoms exist in the form of C-Cl bonds, remaining firmly attached to the carbon atoms and unable to move. However, in a moist state, C-Cl transforms into chloride ions (Cl^-^) that can freely move in the solution. Furthermore, both N and O in CMC-C can form hydrogen bonds with water. Since H_2_O is a polar molecule, charge transfer occurs during hydrogen bond formation. When Cl in the form of an ion carries an electron and diffuses into the solution, the exposed carbon atoms gain 0.041 e, while the connected water molecules gain 0.185 e. Due to the lower electronegativity of nitrogen compared to oxygen, when H_2_O forms hydrogen bonds with N, it gains 0.024 e, whereas CMC-C’s O atom loses only 0.003 e when it forms hydrogen bonds with H_2_O. After the three water molecules adsorb onto CMC-C, 0.204 e from CMC-C is transferred to the water. The adsorption of water molecules onto different aerogel components also triggers different charge transfers. For example, when adsorbed on SA (Fig. 1c), due to its high content of -COONa groups, it loses 0.311 e. In its dry state, sodium atoms on the alginate surface are fixed by oxygen atoms, but when water molecules adsorb, sodium spontaneously forms Na^+^ ions, which diffuse into the solution, generating a large number of -COO^-^ groups that form hydrogen bonds with water (-O…H-O). As a result, electrons transfer from water to sodium alginate, with the adsorbed water molecules carrying + 0.112 e, + 0.108 e, and + 0.88 e of positive charge, respectively. Significant charge transfer also occurs after water molecules adsorb onto the surfaces of SH (Figure S11a, SI) and poly(sodium 4-styrenesulfonate) (Figure S11b, SI). On the surface of sodium hyaluronate, two oxygen-containing groups, -COONa and -OH, both interact with water molecules and facilitate charge transfer. After two water molecules adsorb, sodium hyaluronate carries -0.049 e of negative charge, while the water molecules carry + 0.028 e and + 0.024 e of positive charge. On the surface of poly(sodium 4-styrenesulfonate) (PSS), the sulfonic groups (-SO_3_) interact with water molecules, and the three exposed oxygen atoms (from two -S-O and one -S = O groups) form hydrogen bonds with water. This interaction results in a negative charge of -0.195 e on the surface, with the two water molecules interacting with the -S-O groups carrying + 0.070 e and + 0.072 e of positive charge, and the water molecule interacting with the -S = O group carrying + 0.053 e. The charge transfer between cellulose-based composite aerogels and water molecules provides the foundation for HEG current output. Water molecules carrying positive charges may transform into hydronium ions (H_3_O^+^), while water molecules carrying negative charges may transform into hydroxide ions (OH^-^). Under the influence of the electrodes, these ions may undergo directional movement, generating current in the external circuit.

In addition to the differences in charge transfer, the adsorption energy of H_2_O also varies among different components (Table S1, SI). The binding energies between CMC-C and sodium alginate with water molecules are both greater than 0, indicating that H_2_O does not spontaneously adsorb onto them. In contrast, although the binding energies of PSS and sodium hyaluronate with H_2_O are less than 0, meaning H_2_O can spontaneously adsorb onto their surfaces, the absolute values of the binding energies are relatively small, indicating poor ability to adsorb water molecules. Therefore, after preparing the CMC-C composite aerogel, a layer of MG is grown in situ on the surface of CMC-C. Through the high hygroscopicity of MG, it captures H_2_O from the air, which then penetrates into the CMC-C composite aerogel via capillary action. The synergistic effect between MG and CMC-C enhances the frequency of H_2_O adsorption-evaporation, enabling the simultaneous enhancement of heat dissipation and output current density.

## On-chip thermal utilization system electrical performance characterization

**Fig. 2:**
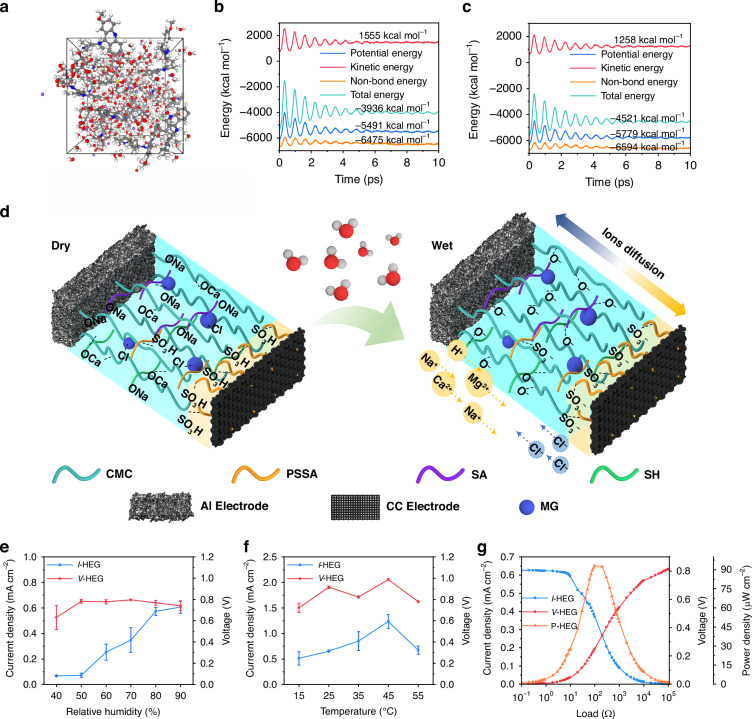
The mechanism analysis and power generation performance characterization. **a** Components of the HEG aerogel. The variations in kinetic energy, potential energy, and total energy of the HEG aerogel at **b** 25 °C and **c** -15 °C. **d** Schematic diagram of the HEG power generation principle. The average I-V curves (current density (blue) and voltage (red)) of a HEG single module over 30 min under **e** different humidity levels and **f** different temperatures, as well as **g** the current density (blue), open-circuit voltage (red), and power density (orange) under different loads. Error bars represent standard deviation

To investigate the interaction between the aerogel and water molecules under different environmental conditions, a aerogel model was constructed based on the molar ratio of the different components of the aerogel, with a total of 200 molecules. Molecular dynamics simulations were performed to observe the movement of the aerogel in different states (Fig. 2a). When the environmental temperature was 25 °C, as water molecules continuously evaporated and diffused within the model, the energy generally showed a downward trend. After 4 ps, the energy fluctuations gradually stabilized, and the kinetic energy remained at 1555 kcal mol^-1^ (Fig. 2b), maintaining dynamic equilibrium. The relatively high kinetic energy indicated that particles in the module were moving vigorously, with frequent adsorption and desorption events between H_2_O and the CMC-C composite aerogel. As the environmental temperature decreased, the module ‘s kinetic energy also decreased. When the temperature dropped to -15 °C, the module ‘s kinetic energy significantly decreased from 1555 kcal mol^-1^ to 1258 kcal mol^-1^ (Fig. 2c), with low temperatures effectively inhibiting interactions between H_2_O and the CMC-C composite aerogel.

Although the kinetic energy of the aerogel was lower at low temperatures than that at higher temperatures, the aerogel also inhibited the freezing of water molecules. By analyzing the bond lengths of the O and H atoms in the aerogel’s movement, the hydrogen bond length in the module remained constant at 2.23 Å, both at room temperature and at low temperatures (Figure S12, SI). Furthermore, by tracking the number of hydrogen bonds in the model at room and low temperatures (Figure S13, SI), it was found that at room temperature, water molecules in the module fluctuated more violently, leading to a smaller number of hydrogen bonds, which fluctuated between 340 and 370. However, as the temperature decreased to -15 °C, the number of hydrogen bonds remained in the range of 350 to 380, slightly higher than at 25 °C. This indicates that water molecules still existed in a liquid or gaseous state. Components such as Na and Cl in the aerogel, upon adsorption of H_2_O, were transformed into Na^+^ and Cl^-^, effectively inhibiting the freezing of water in the aerogel, ensuring that HEG could still function properly under low-temperature conditions.

The mechanism of action of HEG is illustrated in Fig. 2d. When dry, the Cl, Ca, Na, and Mg ions in the aerogel’s complex salts remain stable. Although there is a certain potential difference between the two electrodes, the limited potential difference is insufficient to drive ion dissociation from substances like CMC-C and induce directional movement, meaning no current is generated in the external circuit. When HEG is exposed to a humid environment, it continuously absorbs H_2_O from the surroundings. Under the influence of H_2_O, elements such as Cl, Ca, and Na gradually decompose from the aerogel structure, causing ions to undergo directional movement under the electrostatic forces at the electrodes, generating a detectable electrical signal in the external circuit (Figure S14, SI).

The I-V curves (output current density and open-circuit voltage) of the HEG were tested under various humidity conditions (Fig. 2e). The results indicate that at lower humidity levels, Cl^-^ and Na^+^ cannot fully hydrolyze to form free particles, resulting in a low current density, with the voltage-current density remaining below 0.1 mA cm^-2^ and the open-circuit voltage at only 0.6 V. As humidity increases, the degree of ion hydrolysis in the aerogel increases, leading to a significant rise in current density. At 90% humidity, the current density reaches as high as 0.6 mA cm^-2^. The voltage stabilizes after reaching 0.78 V and does not exhibit significant humidity dependence, further confirming that the voltage is primarily influenced by the potential difference between the electrodes rather than environmental factors. Additionally, since indoor humidity typically ranges from 40% to 60%, the HEG can be applied in various indoor environments while providing stable electrical signals. The changes in current density and voltage at different temperatures further demonstrate that current density is more affected by environmental conditions (Fig. 2f), while voltage exhibits minimal variation under environmental influences. The current density exhibits a trend of first increasing and then decreasing with temperature. At lower temperatures, as the temperature rises, the degree of ion hydrolysis in the gel increases, leading to higher conductivity (Figure S15, SI). Consequently, the current density of the HEG increases. When the temperature reaches 45 °C, the current density peaks at 1.2 mA cm^-2^. However, at higher temperatures, the CMC-C composite aerogel struggles to absorb sufficient H_2_O, as water evaporates before being absorbed, leading to a significant reduction in current density. To further test the operational performance of the HEG device in real-world conditions, the HEG device was placed directly in an indoor environment (27 °C ≤ T ≤ 28 °C, 40% ≤ RH ≤ 45%). The average current density and voltage of the HEG device after half an hour of operation are shown in Figure S16 (SI), where the current density remained at 0.07 mA cm^-2^ and the open-circuit voltage was 0.74 V, both maintaining a high level. This demonstrates that the HEG device can achieve stable electrical signal output without any additional apparatus. Moreover, under the influence of the bimolecular gel, the HEG is able to generate stable electrical signals even in a low-temperature environment. As shown in Figure S17 (SI), the HEG can still produce a current density of 19 μA cm^-2^ and an open-circuit voltage of 0.48 V at -20 °C, demonstrating that the HEG can operate stably within a wide temperature range from -20 °C to 55 °C. The current-voltage output under various loads was tested according to the circuit diagram in Figure S18 (SI). At 25 °C and 100% humidity, the highest output power of 93.14 μW cm^-2^ is achieved with a 99.9 Ω load (Fig. 2g), which is one of the best results compared to currently published research (Table S2, SI).

**Fig. 3:**
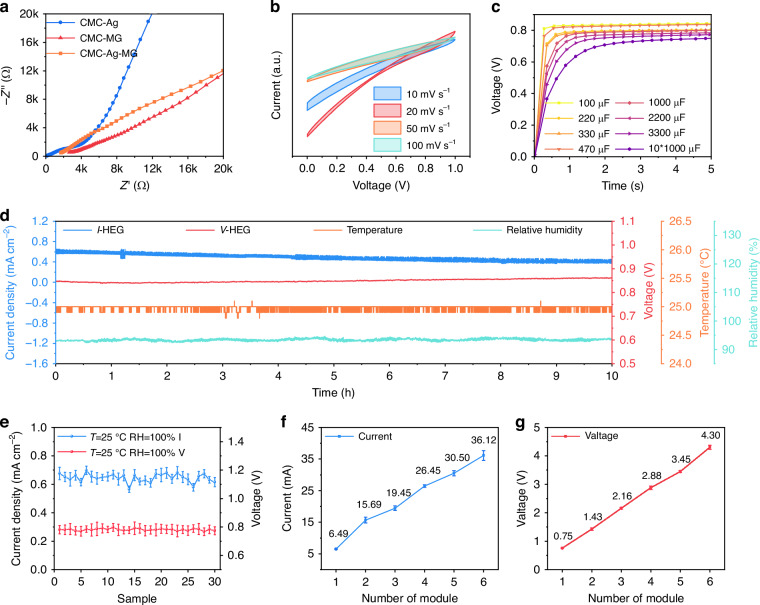
The electrical performance of the HEG. **a** Nyquist of the HEG under different conditions. **b** Cyclic voltammetry (C-V) curves of the HEG. **c** V-t curve of the HEG charging capacitors. **d** Current density (blue) and voltage (red) variation of the HEG over 10 h under conditions of 25 °C and RH = 100%. **e** I-V curves of 30 HEG composite devices. **f** Current variation when 6 HEG modules are connected in parallel. **g** Voltage variation when 6 HEG modules are connected in series. Error bars represent standard deviation

Figure 3a illustrates the effect of different aerogel materials on the impedance of the HEG. Although the addition of Ag and MG leads to an increase in impedance, it effectively enhances the stability and hygroscopic capacity of the device, thereby improving its lifespan and output current density. To investigate the effect of redox reactions on the output electrical signals of the HEG, the relationship between current and potential was tested at different scan rates using cyclic voltammetry (C-V curves) (Fig. 3b). No significant redox peaks were observed, demonstrating that the electrical signals from the HEG originated from the directional movement of ions following hydrolysis in the gel, rather than from corrosion currents generated by the Al electrode. The energy produced by the HEG can be stored in capacitors, with small capacitors below 1 mF being charged within seconds (Fig. 3c), and a large 1.0 F capacitor being fully charged in 20 min (Figure S19, SI). In addition, under high humidity room temperature conditions, the HEG device can operate stably for extended periods (Fig. 3d). After continuous operation for 10 h, it remains stable with no structural failure. To test the stability of the HEG devices, 30 identical HEG modules were prepared using the same method. Observation of the SEM images of the intermediate aerogels reveals that all 30 types of aerogels exhibit similar morphologies, with randomly distributed pores ranging from 80 to 100 μm in diameter within the gel (Figure S20, SI). The gel conductivity also exhibited similar results, with an average conductivity of 2.14 mS m^-1^ for the 30 aerogels under conditions of 25 °C and 100% relative humidity (Figure S21a, SI). When the 30 HEG modules were placed in an environment of 25 °C and 100% relative humidity, the average current density and open-circuit voltage over 30 min, as shown in Fig. 3e, demonstrated good consistency, with a current density of approximately 0.65 mA cm^-2^ and an open-circuit voltage of about 0.78 V. Additionally, significant humidity dependence was observed; in low humidity conditions (RH = 40%), the current density decreased to only 0.065 mA cm^-2^, and the open-circuit voltage dropped to 0.64 V (Figure S21b, SI). This confirms that the preparation of HEG exhibits good reproducibility. Through a simple circuit, the electrical signals output by the HEG modules can be integrated; six devices in parallel can produce a current of 36.12 mA (Fig. 3f), and in series, they can generate a voltage of 4.30 V (Fig. 3g). The amplified circuit after series-parallel connection can directly power timers, electronic watches, and visible light LEDs (Figure S22, SI).

## On-chip thermal utilization system thermal-electric conversion characterization

**Fig. 4:**
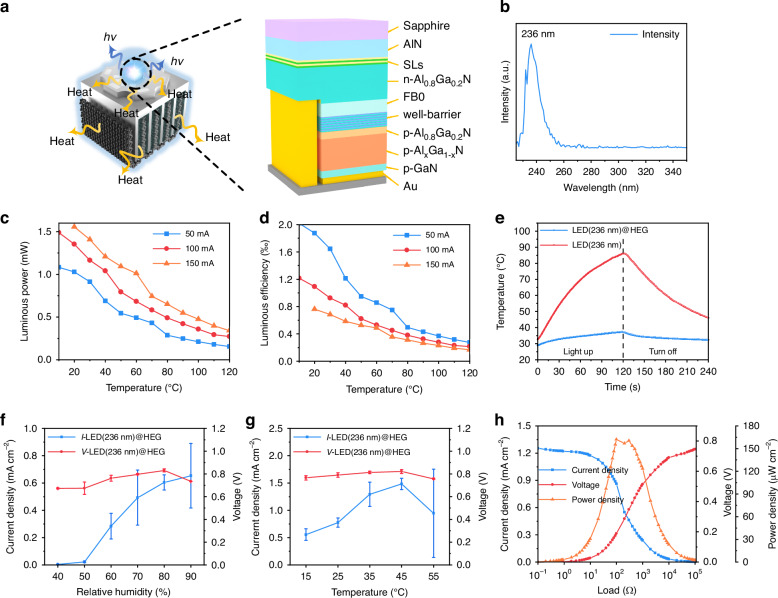
The LED (236 nm)@HEG composite structure and its comprehensive performance. **a** Schematic diagram of the LED (236 nm). **b** Emission intensity as a function of wavelength. **c** Light emission power and **d** electro-optical conversion efficiency of the LED (236 nm) at different currents and temperatures. **e** Temperature variation of the LED (236 nm) (red) and LED (236 nm)@HEG (blue) during continuous operation for 2 min, and temperature variation during a 2 min stop. Testing was conducted in an indoor environment (27 °C ≤ T ≤ 28 °C, 40% ≤ RH ≤ 45%). The LED(236 nm)@HEG device’s average current density (blue) and voltage (red) over 30 min under **f** different humidity levels and **g** different temperatures, as well as **h** the current density (blue), voltage (red), and power density (orange) of the LED(236 nm)@HEG device under different loads. Error bars represent standard deviation

To examine the application prospects of the HEG in the heat dissipation of chips, we fabricated a DUV LED as shown in Fig. 4a. After testing, the central wavelength of the LED was found to be 236 nm (Fig. 4b). As a microelectronic device, the LED is affected by its own wavelength. As the emission wavelength continues to decrease, the electro-optical conversion efficiency typically decreases. During operation a large amount of electrical energy is converted into internal energy, which causes the temperature of the DUV LED to rise continuously. As the temperature increases, the defect concentration in AlGaN rises, leading to enhanced radiative carrier recombination and a continuous decrease in carrier lifetime, which directly results in a reduction in LED light emission intensity^38,39^. Additionally, with the temperature increase, lattice vibrations intensify (Figure S23, SI), causing a decline in carrier mobility, which further contributes to the decrease in light emission efficiency. At low temperatures (T = 10 °C), when the LED (236 nm) is driven by currents of 50 mA, 100 mA, and 150 mA, the luminous power exceeds 1 mW. However, as the temperature increases, the luminous power also decreases. When the temperature exceeds 100 °C, the luminous power drops to below 0.5 mW (Fig. 4c). Furthermore, as the operating time increases, in the absence of a heat dissipation device, the voltage does not exhibit significant changes with increasing temperature when a fixed input current is applied (Figure S24, SI). However, the luminous power continues to decrease, leading the DUV LED into a vicious cycle of temperature rise and decreased electro-optical conversion efficiency (Fig. 4d).

Based on the heat generation of the LED(236 nm) at 20 °C, a finite element model was constructed. The simulations (Figure S25a, SI) show that the temperature of the LED(236 nm) rises to 84.05 °C within 20 s and, with continued heat accumulation, reaches 168.89 °C in 1 min. Such high temperatures directly lead to a decrease in the LED’s electro-optical conversion efficiency and the lifespan of its core semiconductor. Additionally, while theoretical calculations suggest that the device does not fail due to high temperatures, in practical use, temperatures exceeding 100 °C can cause failure of soldering points, ultimately leading to LED failure as temperatures rise. With the integration of the HEG module, the temperature of the LED(236 nm) is significantly reduced, remaining below 51 °C after 1 min (Figure S25b, SI). A comparison of the temperature changes between a standalone LED(236 nm) and the LED(236 nm)@HEG within 1 min (Figure S26, SI) shows that the temperature of the standalone LED increases linearly over time without reaching a stable plateau, whereas the LED(236 nm)@HEG quickly stabilizes after an initial 1.5 s temperature rise. After loading the HEG, energy from the LED chip is gradually transferred to the Al substrate, Al electrodes, and aerogel via solid-state heat transfer, causing a temperature difference to form within the HEG (Figure S27a-c, SI). As the temperature changes, the potential difference across the asymmetric electrodes does not exhibit significant variation (Figure S27d, SI), thus not leading to a notable decrease in the HEG open-circuit voltage. However, fluctuations in temperature directly lead to changes in the degree of ionic hydrolysis. As a result, an ionic concentration gradient is established within the aerogel due to the temperature gradient between the high-temperature Al electrode and the low-temperature CC electrode. Under the influence of the chemical potential difference, ions spontaneously migrate from the region of higher concentration to the region of lower concentration, thereby enhancing the ionic diffusion rate. Moreover, because the distance between the electrodes remains constant, the diffusion distance for the ions does not change. Therefore, the increase in diffusion rate promotes an enhancement in the current density output of the HEG.

The actual testing results also validate the accuracy of the finite element simulation (Fig. 4e). Under indoor conditions (27 °C ≤ T ≤ 28 °C, 40% ≤ RH ≤ 45%), when a current of 100 mA is applied to the LED (236 nm), the temperature of a single LED module (236 nm) reaches 86.01 °C within 2 min. In contrast, when the LED (236 nm) is combined with HEG to form a composite device, the temperature only rises to 37.26 °C after the same duration under identical conditions, which is significantly lower than the temperature of the standalone LED (236 nm) module during operation. Furthermore, using cooling solutions such as microchannels, thermal interface materials (TIMs), heat-spreading composites, and vapor chambers, the temperature of the LED (236 nm) exceeds 50 °C after 2 min of operation (Figure S28, SI). Although this represents a marked reduction compared to the standalone LED (236 nm), it still fails to achieve the cooling effectiveness seen with HEG. The existing technology requires active cooling methods to maintain the temperature of the LED (236 nm) below 45 °C (Figure S29, SI). Using thermoelectric cooling (TC) technology, the temperature can be controlled at 42.57 °C with an input of 3.3 W of electrical power to drive the TC. In contrast, employing a water microchannel necessitates 3.0 W to drive a peristaltic pump, which can reduce the LED (236 nm) temperature to 37.26 °C. However, active cooling demands additional energy input into the system, leading to greater energy waste. Furthermore, the water microchannel requires extra storage space for cold water, which exacerbates its applicability in the microelectronics field. In comparison, HEG achieves temperature control simply through connectivity, significantly saving space and energy, as detailed in Table S3 (SI). Not only can the HEG achieve temperature control of the LED (236 nm) in an exposed environment, but it can also ensure stable operation of the LED (236 nm) at optimal temperatures in a confined sealed environment. When the LED (236 nm)@HEG device is placed inside a sealed electronic enclosure, the HEG continues to generate electrical signals stably, and the confined space promotes moisture circulation, resulting in larger electrical signals compared to those produced in a typical indoor environment (Figure S30, SI). However, the limited environment hinders heat dissipation; after 30 min of operation, the temperature of the LED (236 nm) reached 60 °C, which is still lower than the 80 °C observed for a single LED (236 nm) module operating for 2 min in a natural environment. This demonstrates that the HEG can reliably produce electrical signals in a closed environment while maintaining stable thermal performance.

Additionally, when the LED (236 nm) stopped emitting light, the LED (236 nm)@HEG composite device returned to room temperature within 2 min, while the temperature of the single LED module (236 nm) remained above 45 °C (Video S1, SI). This indicates that the single LED module (236 nm) is difficult to reuse in a short period of time. Compared to the standalone LED(236 nm) module, the LED(236 nm)@HEG exhibits significantly improved lifespan and luminous power. To further explore the potential of the LED(236 nm)@HEG in practical applications, a large-scale integrated model was constructed by integrating 25 LED(236 nm) on a 4×4 cm Al substrate, using 4 HEG modules for heat dissipation (Figure S31, SI). The results shown in Figure S32 (SI) indicate that with 4 HEG modules, the temperature of the 25 LED(236 nm) modules can be reduced from 432.3 °C to 74.0 °C.

After the HEG forms a composite device with the LED (236 nm), not only is the temperature of the LED (236 nm) significantly controlled, but the performance of the HEG is also further enhanced due to the thermal energy generated by the LED (236 nm). Under low humidity conditions (Fig. 4f), due to the influence of the LED temperature, the HEG has difficulty directly capturing water molecules from the air, resulting in a current density decrease of only 0.0039 mA cm^-2^. However, under high humidity conditions, the ion flow rate increases with the temperature, significantly enhancing the current density (Fig. 4g). At RH = 100%, the optimal current density reaches 1.25 mA cm^-2^. The higher current density also facilitates an increase in output power (Fig. 4h), with the LED (236 nm)@HEG device achieving an optimal output power of 162.08 μW cm^-2^, a 74% improvement over the single HEG unit.

**Fig. 5:**
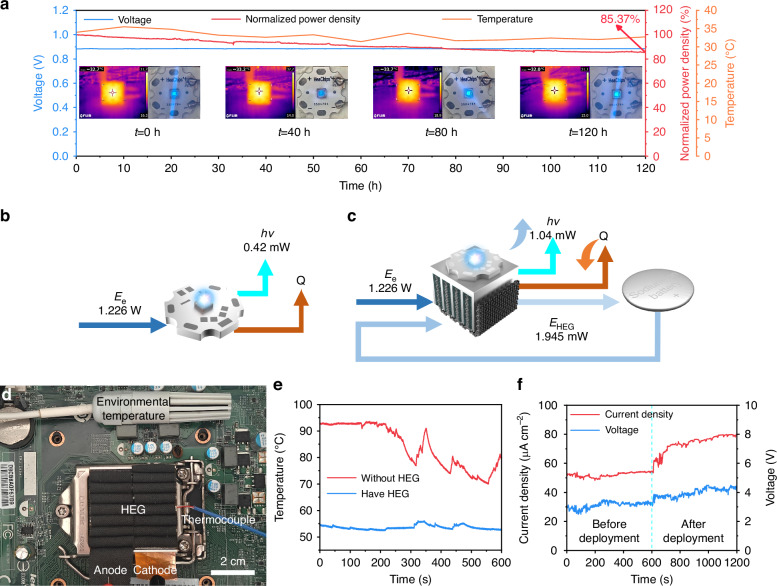
Stability testing and energy cycling. **a** The power generation, heat dissipation, and light emission of the LED(236 nm)@HEG composite device over a 120 h period. Schematic diagram of **b** LED and **c** LED(236 nm)@HEG input energy and its variations. **d** Digital picture of the G3220 chip surface after the deployment of HEG. **e** Temperature variation curve of the chip over time under normal operating conditions. Blue represents the temperature without HEG deployment, and red represents the temperature with HEG deployed. **f** Variation curves of current density (blue) and open-circuit voltage (red) generated by HEG over time

To further verify its stability, the LED (236 nm) @ HEG device was placed in a constant temperature and humidity chamber (T = 20 ± 0.5 °C, RH = 95 ± 3%) and continuously operated for over 120 h (Fig. 5a). The LED temperature remained consistently below 35 °C throughout the 120 h period. After 120 h of testing, the normalized power density of the LED was still maintained at a high level of 0.85. Furthermore, the HEG device maintained a high output voltage of 0.9 V, further confirming the stability of the LED (236 nm) @ HEG composite device.

The power generated by the HEG is not enough to drive an LED(236 nm). However, the energy produced by the HEG can be stored in energy storage devices and then used to supply power to high-power electronic devices when needed, achieving indirect power supply. In terms of LED thermal management, after charging a 1.0 F capacitor with 6 LED(236 nm)@HEG units, it is possible to drive a small fan (Video S2, SI), thereby further controlling the temperature of the LEDs. Additionally, batteries can be utilized as energy carriers for indirect power supply. Following the method outlined in Figure S33 (SI), a battery was fabricated with MoS_2_ as the cathode and a Na sheet as the anode, providing a stable discharge platform for electrical output. Moreover, batteries can be arranged in series-parallel configurations to form battery packs according to different requirements, addressing the issue of capacitors struggling to increase output voltage. As shown in Figure S34 (SI), the LED@HEG device powers a sodium-ion battery, which is then connected in series to form a large battery pack that can subsequently power the LED again, realizing an energy cycle. By using 6 LED(236 nm)@HEG devices to charge 6 batteries, and then connecting the 6 batteries in series, the LED is successfully illuminated (Video S3, SI), thus achieving an energy cycle.

As shown in Fig. 5b, when an input of 1.226 W of electrical energy is provided to the single LED (236 nm) module, it is converted into light and thermal energy. After continuous operation for 2 min, the heat from the LED (236 nm) accumulates, causing the temperature to rise and resulting in a light output of only 0.42 mW, while the remaining 1225.58 mW of energy is dissipated as thermal energy into the environment (Figure S35a, SI). In contrast, the LED(236 nm)@HEG composite device can convert excess thermal energy into electrical energy, feeding it back into the system to create a closed-loop cycle (Figure 35b, SI). With the same input of 1.226 W of electrical energy, the thermal accumulation in the LED (236 nm) is significantly alleviated, allowing for the generation of 1.04 mW of light energy, while the HEG converts 1.945 mW of thermal energy into electrical energy, resulting in a total power output of 2.98 mW (Fig. 5c). Compared to the single LED (236 nm), which only produces 0.42 mW of light energy, the energy utilization efficiency has improved by 610.70% (Note S1, SI).

Thermal energy utilization was also achieved on the commercial Intel G3220 chip (Figure S36a, SI). This was accomplished by simply attaching a device formed by connecting four HEG units in series to the surface of the chip (Fig. 5d). When the chip was directly exposed to the indoor environment (27 °C ≤ T ≤ 28 °C, 40% ≤ RH ≤ 45%), under extreme conditions, the CPU’s processing rate was limited to 1.45 GHz at full power output due to heat generation, and the CPU usage was only 48.5% (Figure S36b, SI). However, after integrating the HEG device, the processing rate increased by 16.6%, reaching 1.69 GHz, and the CPU usage rose to 56.9% (Figure S36c, SI). During normal use, the temperature of the individual CPU reached 93 °C, while the temperature dropped to below 60 °C with the HEG device integrated (Fig. 5e). Additionally, the heat generated by the chip exacerbated the ion concentration gradient between the two electrodes, resulting in an open-circuit voltage increase from approximately 3.2 V to 4.2 V. As the temperature increased, the degree of ion hydrolysis intensified, carrier concentration increased, and current density rose from 53 μA cm^-2^ to 79 μA cm^-2^ (Fig. 5f).

To further validate the thermal control capability of HEG for electronic devices and its universality in the field of thermal management, we tested the temperature variations of HEG under different heat flux densities (Figure S37a, SI). At a high heat flux density of 11,250 W m^-2^, the standalone HEG device maintained a temperature below 70 °C, ensuring that heating electronic devices could operate continuously at favorable temperatures. This was also demonstrated in applications involving commercial high-power LEDs (Figure S37b-e, SI), where the temperature exceeded 80 °C when the LEDs were used alone. Notably, after 2 min of operation, the temperatures of the red and green LEDs even surpassed 100 °C. In contrast, when integrated into a composite device with HEG, the temperature could be controlled to remain below 40 °C. The HEG also exhibited a measurable temperature control effect on motors with irregular surface morphologies, resulting in a temperature decrease of 16 °C after 2 min of operation (Figure S37f, SI). When using an RFA as the heat source (Figure S37g, SI), six HEG modules were arranged on the surface of the RFA, taking into account the actual dimensions of the RFA (Figure S37h, SI). By comparing the temperature changes before and after the application of the composite material (Figure S37i, SI), the temperature decreased from 56.42 °C to 33.43 °C after 2 min. This demonstrates the versatility of HEG in thermal energy control and its effectiveness in the utilization of thermal energy.

## Discussion

In summary, we have developed a thermal energy utilization device based on the HEG and successfully applied it to chips with varying power levels, achieving both heat absorption and conversion. When the chip is idle, the HEG absorbs heat and water molecules from the surrounding environment, generating a power density of 0.61 mA cm^-2^, an open-circuit voltage of 0.78 V, and a peak power of 91.14 μW cm^-2^. When integrated with the high-heat generating DUV LED (236 nm), it significantly controls the temperature of the LED (236 nm) and increases the overall energy utilization efficiency from 0.42 mW to 2.98 mW. The generated electrical energy, using a sodium-ion battery as a medium, can be used to power the LED (236 nm). By utilizing series or parallel configurations, the device’s output current and voltage can be enhanced. On the commercial Intel G3220 chip, we successfully increased the chip frequency by 16.6% and converted thermal energy into electrical energy storage. The temperature variations under different heat flux densities, along with the successful applications in high-power LEDs, motors, and radio frequency amplifiers, also demonstrate the universality of HEG in the thermal utilization of electronic devices.

## Materials and Methods

Preparation of CMC-C Aerogel Precursor 0.5 g of CMC-C and 0.2 g of MWCNTs were sequentially added to 10 mL of a low molecular weight sodium hyaluronate solution (0.1 g/mL in water) while continuously stirring, referred to as Solution A. Subsequently, 1.0 g of CMC-C and 1.0 g of sodium alginate were added to 10 mL of dimethyl sulfoxide (DMSO) with stirring throughout the addition process, referred to as Solution B. After stirring for 1 h, 0.2 g of PSS was added to Solution A. Then, Solution B was poured into Solution A, and the mixture was heated in a water bath at 50 °C for 6 h to obtain the CMC-C aerogel precursor.

Preparation of HEG Devices Due to the irregular multi-fin structure of the Al electrodes, five fins measuring 20 × 1 × 14 mm were evenly distributed on a 20 × 20 × 2 mm Al substrate, with a spacing of 3.75 mm between each fin. The CMC-C aerogel precursor was then completely filled into the gaps between the fins, and a CC electrode was inserted into the center of the gel. The filled HEG precursor module containing CMC-C aerogel and the CC electrode was then placed in an environment at -20 °C to freeze overnight. Subsequently, it was freeze-dried under conditions of -18 °C and 1 mbar for 48 h. Following this, the HEG module was removed and immersed in a 2 wt% CaCl_2_ aqueous solution for 12 h, after which it was transferred to an MG precursor solution for 1 h. Finally, the HEG module was dried overnight at 70 °C. After testing, the thermal conductivity of the aerogel was found to be 7.11 W (m·K)^-1^. Given that the Al electrodes contain four voids, each HEG module comprises four HEG units, with each unit sized at 20 × 4.75 × 14 mm, while the overall dimensions of the module are 20 × 20 × 16 mm. When using HEG for cooling heat-generating devices, a thermal adhesive is applied to attach the HEG to the surface of the heating device. For further details on materials and preparation methods, see Note S2 (SI).

Finite Element Simulation The simulation was carried out using the heat transfer module in COMSOL Multiphysics 6.0. The material was defined and simulated based on solid heat transfer and shell heat transfer. First, a 3D model was created based on the dimensions of the LED and HEG, followed by the assignment of material properties according to the actual composition of the materials in the model. All surfaces were set to be in contact with air. During the simulation, the mesh was controlled by the physical field, with extremely fine elements. The transient calculation was then performed to obtain the temperature change of the model over time.

First-Principles Calculations First-principles calculations and visualization were performed using Materials Studio 2020. The simulations based on first-principles calculations mainly included the variation of molecular surface charge density, the binding energy between the aerogel matrix and water molecules, and molecular dynamics calculations. The molecular surface charge density and binding energy with water molecules were calculated using the DMol 3 module^40–42^. The modeling was based on the actual structure of different molecules, with polymer molecules being simulated by monomers. The calculation precision was set to “Fine.” After morphological optimization, “Energy” was used to calculate the surface charge density and energy within the module. The binding energy was determined by comparing the module’s energy with and without water. Molecular dynamics were calculated using the Forcite module, with temperature control applied to the molecular fluctuations^43,44^. The calculation precision was set to “Ultra-fine,” and the molecular motion was calculated over a period of 10 ps, with a result output every 0.1 ps.

Preparation of sulfur ion batteries In order to evaluate the performance of the battery, 2032-coin cells are assembled^45^. N-methyl-2-pyrrolidone (NMP) is employed as the organic solvent, and the active materials, Super-P and PVDF binder, are combined in a ratio of 7:2:1 by mass. The slurry is applied to a pure copper foil in order to prepare the working electrode. A pure sodium sheet is employed as the counter electrode, while a glass microfiber filter (GF/D) is utilized as the separator. The electrolyte was 1.0 M LiPF₆ dissolved in a mixture of ethylene carbonate and dimethyl carbonate in a 1:1 volume ratio.

Calculation method for the overall efficiency of the LED@HEG composite device. The input electrical energy of the LED is primarily converted into thermal energy and optical energy. Therefore, the LED electro-optical conversion efficiency can be expressed by the following formula:

$${W}_{E}={W}_{L}+{Q}_{H}$$

$${\eta }_{L}=\frac{{W}_{L}}{{W}_{E}}$$

Where *W*_*E*_ represents the total energy input to the system (electrical power), *W*_*L*_ represents the output optical power, *Q*_*H*_ represents the output thermal power, and *η*_*L*_ represents the LED electro-optical conversion efficiency.

As the usage time increases, the temperature of the LED continues to rise, while the luminous efficiency gradually decreases. Therefore, the luminous efficiency and energy conversion efficiency at a specific time point can be expressed by the following formula:

$${W}_{E}^{t}={W}_{L}^{t}+{Q}_{L}^{t}$$

$${\eta }_{L}^{t}=\frac{{W}_{L}^{t}}{{Q}_{L}^{t}}$$

Where ${W}_{E}^{t}$ represents the input electrical power at a specific time point, ${W}_{L}^{t}$ represents the output optical power at a specific time point, ${Q}_{L}^{t}$ represents the output thermal power at a specific time point, and ${\eta }_{L}^{t}$ represents the LED electro-optical conversion efficiency at a specific time point.

After the formation of the composite device between HEG and the LED (236 nm), the input electrical power is not only converted into optical energy but also captured by the HEG and re-converted into electrical energy. Therefore, the energy utilization efficiency and conversion rate at a specific time point can be expressed by the following formula:

$${W}_{all}^{t}={W}_{L}^{t}+{W}_{H}^{t}$$

$${\eta }_{all}^{t}=\frac{{W}_{all}^{t}}{{W}_{E}^{t}}$$

Where ${W}_{all}^{t}$ represents the output power of the LED (236 nm)@HEG composite device at a specific time point, ${W}_{H}^{t}$ represents the generated electrical energy (output power) of the HEG at the specific time point, and ${\eta }_{all}^{t}$ represents the energy conversion efficiency of the LED (236 nm)@HEG composite device.

## Supplementary information

Supplementary Information for Thermal Utilization on Chip

Temperature variation of the LED(236 nm) module, LED(236 nm)@HEG device

LED(236 nm)@HEG driving a small fan

LED(236 nm)@HEG driving a LED

## Data Availability

All data generated or analyzed during this study are included in this published article (and its Supplementary Information files).
