# Supplementary material for: Thermal Utilization on Chip
Source: Light Sci Appl. 2026 Jun 2;15:261. doi: 10.1038/s41377-026-02326-1 (PMC13230715; doi:10.1038/s41377-026-02326-1)
Supplement: Supplementary file 1 — Supplementary Information for Thermal Utilization on Chip [file 41377_2026_2326_MOESM1_ESM.docx]

Supplementary Information for

**Thermal Utilization on Chip**

Yaohao Zhang^1,2 #^, Bo Lai^1 #^, Fei Yu^2^, Xuesong Li^2^, Yue Yang^2^, Wei Lü^1,2 *^, Ke Jiang^1^, Xiaojuan Sun^1 *^, and Dabing Li^1,3 *^

^1^State Key Laboratory of Luminescence Science and Technology, Changchun Institute of Optics, Fine Mechanics and Physics, Chinese Academy of Sciences, Changchun 130033, China

^2^Key Laboratory of Advanced Structural Materials, Ministry of Education and School of Materials Science and Engineering and Advanced Institute of Materials Science, Changchun University of Technology, Changchun, 130012, China.

^3^Center of Materials Science and Optoelectronics Engineering, University of Chinese Academy of Sciences, Beijing 100049, China

* Corresponding author (s): [lw771119@hotmail.com](mailto:lw771119@hotmail.com) (W. Lü); [sunxj@ciomp.ac.cn](mailto:sunxj@ciomp.ac.cn) (X. Sun); [lidb@ciomp.ac.cn](mailto:lidb@ciomp.ac.cn) (D. Li).

Fax: +86-0431-85716426; Tel: +86-0431-85716421

**Content**

[Supplementary Notes 4](#_Toc227483761)

[Note S1 Calculation method for the overall efficiency of the LED@HEG composite device. 4](#_Toc227483762)

[Note S2 Materials and preparation methods. 5](#_Toc227483763)

[Supplementary Figure 8](#_Toc227483764)

[Figure S1 Flowchart of the HEG module preparation method. 8](#_Toc227483765)

[Figure S2 Characterization of Al@Al(OH)_3_ composite electrode.. 9](#_Toc227483766)

[Figure S3 Digital photograph of the HEG device under a pressure of 4 MPa. 10](#_Toc227483767)

[Figure S4 SEM images of ZIF67@CC.. 11](#_Toc227483768)

[Figure S5 Characterization of ZIF67@CC composite electrode.. 12](#_Toc227483769)

[Figure S6 Infrared Fourier transform spectra of ZIF67@CC composite electrode. 13](#_Toc227483770)

[Figure S7 Differential charge density of different electrode materials.. 14](#_Toc227483771)

[Figure S8 Infrared Fourier transform spectra of CMC-C gel composite before and after MG. 15](#_Toc227483772)

[Figure S9 Characterization of gel.. 16](#_Toc227483773)

[Figure S10 CMC-C gel water absorption capacity.. 17](#_Toc227483774)

[Figure S11 Simulation of surface charge of gel monomers.. 18](#_Toc227483775)

[Figure S12 The bond length distribution of O-H at different temperatures.. 19](#_Toc227483776)

[Figure S13 The variation in the number of hydrogen bonds in the gel box at different temperatures. 20](#_Toc227483777)

[Figure S14 Equivalent circuit diagram for load testing.. 21](#_Toc227483778)

[Figure S15 Graph of the conductivity of cellulose-based aerogels as a function of temperature. 22](#_Toc227483779)

[Figure S16 Average current density and voltage of the HEG in a closed environment during the on-off state of the LED (236 nm) over a period of 30 min.. 23](#_Toc227483780)

[Figure S17 The I-V curve of the HEG device over a 30 min period in a low-temperature environment. 24](#_Toc227483781)

[Figure S18 Equivalent circuit diagram for load testing. 25](#_Toc227483782)

[Figure S19 Voltage variation during the charging of a 1.0 F capacitor using the LED@HEG composite device. 26](#_Toc227483783)

[Figure S20 30 different aerogel SEM images. 27](#_Toc227483784)

[Figure S21 Reproducibility testing of 30 HEG devices. 28](#_Toc227483785)

[Figure S22 Digital photos of the composite device during actual usage.. 29](#_Toc227483786)

[Figure S23 First-principles-based simulation of AlGaN. 30](#_Toc227483787)

[Figure S24 LED (236 nm) electrical performance characterization. 31](#_Toc227483788)

[Figure S25 Thermal finite element simulation. 32](#_Toc227483789)

[Figure S26 Temperature variation of the LED(236 nm) before and after forming a composite device with the HEG. 33](#_Toc227483790)

[Figure S27 Temperature finite element simulation and electrode thermal performance characterization. 34](#_Toc227483791)

[Figure S28 The temperature variation of the LED (236 nm) with the use of passive cooling methods. 35](#_Toc227483792)

[Figure S29 The temperature control of the LED (236 nm) using active cooling methods. 36](#_Toc227483793)

[Figure S30 Performance testing of the LED (236 nm)@HEG composite device in a closed environment. 37](#_Toc227483794)

[Figure S31 Finite element analysis of large integrated devices.. 38](#_Toc227483795)

[Figure S32 Temperature variation of the 25 LED(236 nm) before and after forming a composite device with the 4 HEG. 39](#_Toc227483796)

[Figure S33 Schematic diagram of the sodium-ion battery structure. 40](#_Toc227483797)

[Figure S34 Schematic diagram of energy cycling. 41](#_Toc227483798)

[Figure S35 Energy flow diagrams. 42](#_Toc227483799)

[Figure S36 Application of HEG on the Intel G3220 chip. 43](#_Toc227483800)

[Figure S37 HEG Cooling Performance Testing. 44](#_Toc227483801)

[Supplementary Table 45](#_Toc227483802)

[Table S1 The binding energy of different components of the composite gel with H_2_O. 45](#_Toc227483803)

[Table S2 Comparison of device output current, voltage, and optimal power.^1-12^ 46](#_Toc227483804)

[Table S3 Comparison of the Effects of Different Cooling Methods 48](#_Toc227483805)

[Supplementary Video 49](#_Toc227483806)

[Video S1 Temperature variation of the LED(236 nm) module, LED(236 nm)@HEG device. 49](#_Toc227483807)

[Video S2 LED(236 nm)@HEG driving a small fan. 49](#_Toc227483808)

[Video S3 LED(236 nm)@HEG driving a LED. 49](#_Toc227483809)

[References 50](#_Toc227483810)

# Supplementary Notes

## Note S1 Calculation method for the overall efficiency of the LED@HEG composite device.

After the LED (236 nm) operates continuously for 2 min, its temperature rises to 86.01 ℃, which can be approximated as 90 ℃, at which point the luminous power of the LED (236 nm) is 0.42 mW. In contrast, after the LED (236 nm)@HEG composite device operates continuously for 2 min, its temperature is only 37.26 ℃, which can be approximated as 40 ℃, and the luminous power of the LED (236 nm) is 1.04 mW. Under the influence of the LED (236 nm), the HEG's optimal power generation per unit area is 162.08 μW cm^-2^. With an effective area of 12 cm^2^ for a single HEG device, the HEG's power generation is 1.94 mW. The combined energy utilization power of the LED (236 nm)@HEG composite device is 2.98 mW.

## Note S2 Materials and preparation methods.

**2.1 Materials**

Calcium carboxymethyl cellulose (CMC-C, 99%, C922376), ethanolamine (99%, E808764), sodium alginate (SA, High viscosity type Ⅱ, 1% viscosity: 7000-10000 mPa·s, H875028), ethyl acetate (EAC, 99%, E809174), poly(ethylenesulfonic acid) (PSS, average Mw ~70000, Powder, P874964), 2-methylimidazole (98%, Powder, M915172), and magnesium chloride (MgCl_2_, 99%, Powder, M813763) are all sourced from Shanghai Macklin Biochemical Technology Co., Ltd. Dimethyl sulfoxide (DMSO, >99%, D103272), and sodium hydroxide (NaOH, 96%, S111518) are all sourced from Shanghai Aladdin Biochemical Technology Co., Ltd. Low molecular weight sodium hyaluronate (J201231021) is sourced from Bloomage Biotechnology Co., Ltd. Ethanol (CH_3_CH_2_OH, ≥99.7%), methanol (CH_3_OH), calcium chloride (CaCl_2_, AR, 20191009), and hexahydrated cobalt nitrate (Co(NO_3_)_2_·6H_2_O, ≥98.5 %) are all sourced from Sinopharm Chemical Reagent Co., Ltd. Polyvinylidene fluoride (PVDF, HSV900) is sourced from Arkema S.A. (ARKAY). Polydimethylsiloxane (PDMS, DC184) is sourced from Dow Corning. Unless otherwise specified, the H_2_O resistance used in the experiment exceeded 18.2 MΩ·cm^-1^.

**2.2 The oxidation of carbon cloth (CC) and carbon nanotubes (CNTs)**

Firstly, concentrated H_2_SO_4_ concentrated HNO_3_ are mixed in a 3:1 volume ratio and then poured into a beaker containing CC or CNTs. After allowing the mixture to stand for 48 h, the material is removed and repeatedly rinsed with deionised water until the pH becomes neutral. The material is then dried in an oven at 60 ℃ for 24 h.

**2.3 Preparation of ZIF67@CC Composite Electrode**

Firstly, ZIF-67 nanoparticles are synthesized. 0.58 g of Co(NO_3_)_2_·6H_2_O and 3.2 g of 2-methylimidazole are separately placed into two beakers, each containing 40 mL of methanol. Once the powders are completely dissolved, the two solutions are rapidly mixed and stirred vigorously for 2 h. The resulting mixture is then centrifuged to separate the powder, which is washed repeatedly with ethanol and centrifuged three times. Finally, the powder is dried overnight at 60 ℃.

Next, a ZIF-67 spray solution is prepared. PVDF solution (0.3 wt% in EAC) and PDMS solution (22 mg/mL in EAC) are mixed in a 4:6 volume ratio. 0.05 g of ZIF-67 powder is added to 10 mL of the mixed solution and sonicated to ensure complete dispersion, resulting in the ZIF-67 spray solution.

To form the composite electrode, CC is placed on a heated platform at 50 ℃. The spray solution is evenly applied to the surface of the CC at 10 MPa. The coated CC is then kept on the heated platform for 30 min for preliminary curing, before being transferred to an oven at 100 ℃ to cure for 2 h.

**2.4 Pre-treatment of Al Electrode**

Firstly, the surface of the Al electrode is cleaned with alcohol to remove impurities. The Al electrode is then immersed in a NaOH solution (0.05 M in H_2_O) and subjected to a water bath at 80 ℃ for 5 min. Afterward, the Al electrode is quickly transferred into boiling H_2_O and soaked for 30 min. During this process, Al(OH)_3_ grows on the surface of the Al electrode, transforming the surface from hydrophobic to superhydrophilic.

**2.5 Synthesize of MoS_2_**

The layered MoS_2_ is prepared via a one-step hydrothermal method by dissolving (NH_4_)_6_Mo_7_⋅4H_2_O and CH_4_N_2_S in 30 mL of distilled water. The solution is stirred at 25 ℃ for 2 h, then transferred to a 40 mL high-pressure sterilization reactor and maintained in a hydrothermal oven at 180 ℃ for 24 h. After cooling to room temperature, the product was washed several times with distilled water and anhydrous ethanol. It is then dried at 60 ℃ for 12 h and used for subsequent characterization tests.

**2.6 Synthesize of MnSe@MoS_2_**

First, MnO_2_ is prepared through a hydrothermal method by dissolving an appropriate amount of KMnO_4_ in 40 mL of DI water, stirring at room temperature for 30 min until the solution was complete. The solution was then transferred to a Teflon high-pressure reactor and hydrothermally treated at 150 ℃ for 12 h. After cooling to room temperature, the product was filtered, washed, and dried. An appropriate amount of MnO_2_ and Se powder was placed in a tube furnace filled with Ar and heated at 350 ℃ for 2 h. Finally, MnSe@MoS_2_ is prepared by adding the powdered MnSe to the previously synthesized MoS_2_ solution and hydrothermally reacting at 180 ℃ for 24 h.

**2.7 Synthesize of LED (236 nm)**

The epitaxial structure of the LED (236 nm) are grown by a high-temperature metal-organic chemical vapor deposition (MOCVD) system on 2 inch high-temperature annealed (HTA) c-oriented sputtering AlN/Sapphire substrates with 200 nm AlN layer. TMAl and TMGa are used as the metal-source, NH_3_ is used as the N-source, Cp_2_ Mg is used as the Mg-source for p-type doping, SiH4 is used as Si-source for n-type doping, and H_2_ is used as the carrier gas. The epitaxial structure consists of a 150 nm AlN, a 90 nm AlGaN/AlN superlattice stress relaxation layer, a 1000 nm n-Al0.8Ga0.2N layer, a 37 nm i-type Al0.8Ga0.2N barrier layer (First Barrier, FB0), a 3 nm Al0.7Ga0.3N/4 nm AlN MQW layer (total of 6 layers.), a 20 nm p-Al0.8Ga0.2N hole injection layer, a 90 nm p-Al0.8Ga0.2N to p-GaN gradient layer, and a 20 nm p-GaN contact layer, sequentially

**2.8 Testing Methods**

The surface morphology of the device is observed using a scanning electron microscope (SEM). The material's phase information is characterised using an X-ray diffraction (XRD) instrument. X-ray photoelectron spectroscopy (XPS) is employed to examine the electronic states of different elements in the gel. The internal functional groups of the gel are characterised by infrared (IR) absorption spectroscopy. The pore size distribution of the gel is determined using a nitrogen adsorption apparatus. The surface temperature of the device is monitored using an infrared thermal imaging camera. A constant temperature and humidity environment is maintained using a temperature and humidity chamber. The electrical signal variations of the device are measured using a digital source meter. The capacitance-voltage (C-V) curve and impedance spectrum of the device are recorded using an electrochemical workstation.

# Supplementary Figure


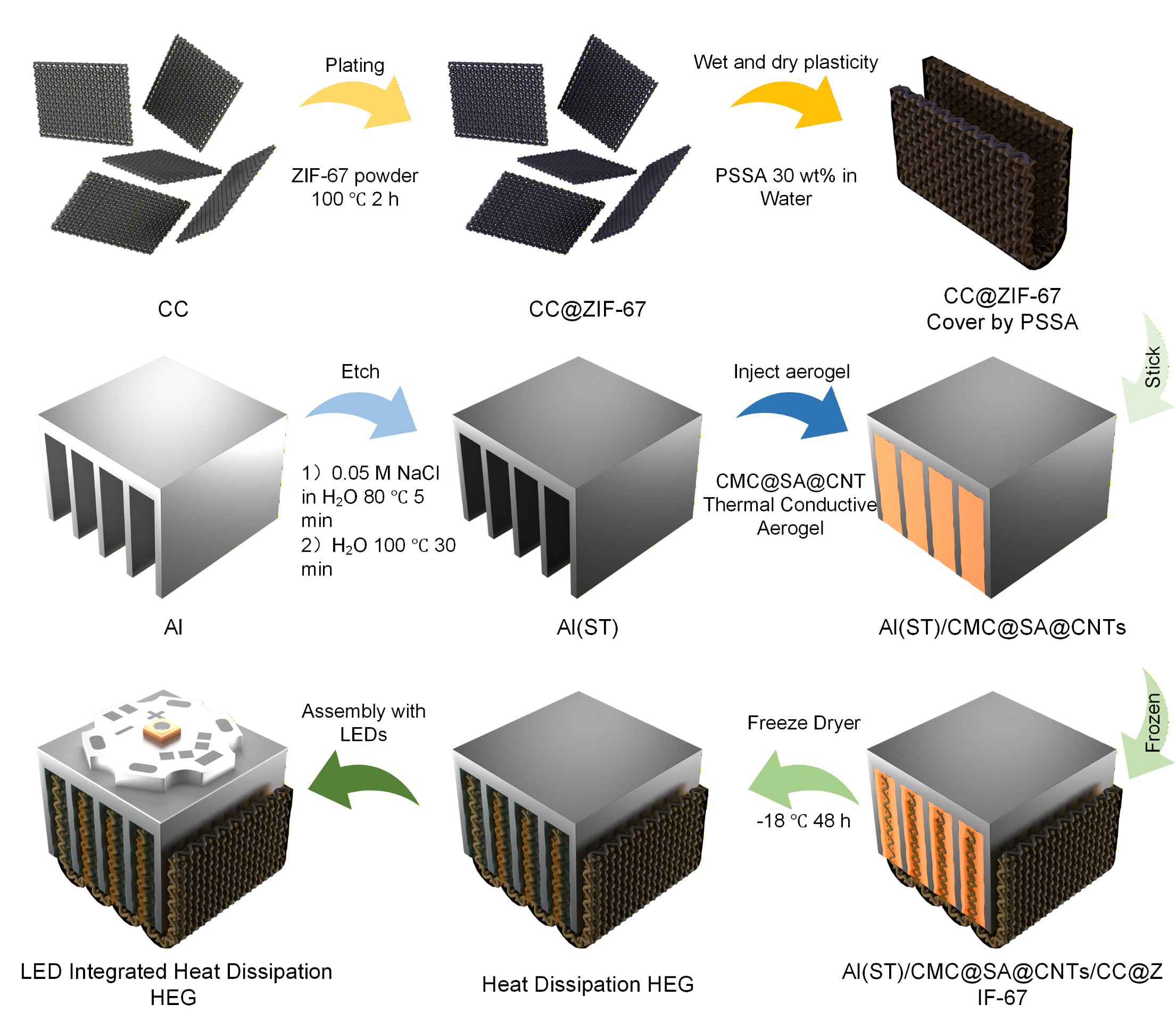


## Figure S1 Flowchart of the HEG module preparation method.


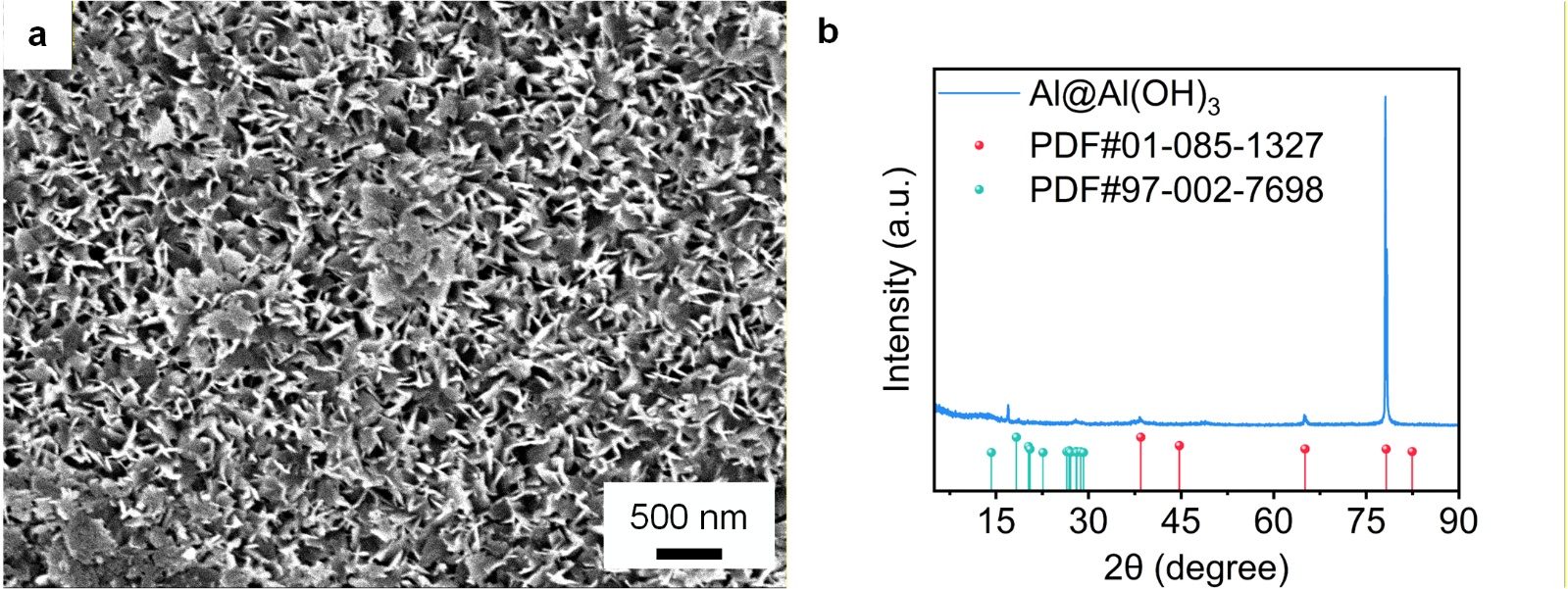


## Figure S2 Characterization of Al@Al(OH)_3_ composite electrode. (a) SEM image and (b) XRD spectrum of the Al composite electrode.

**
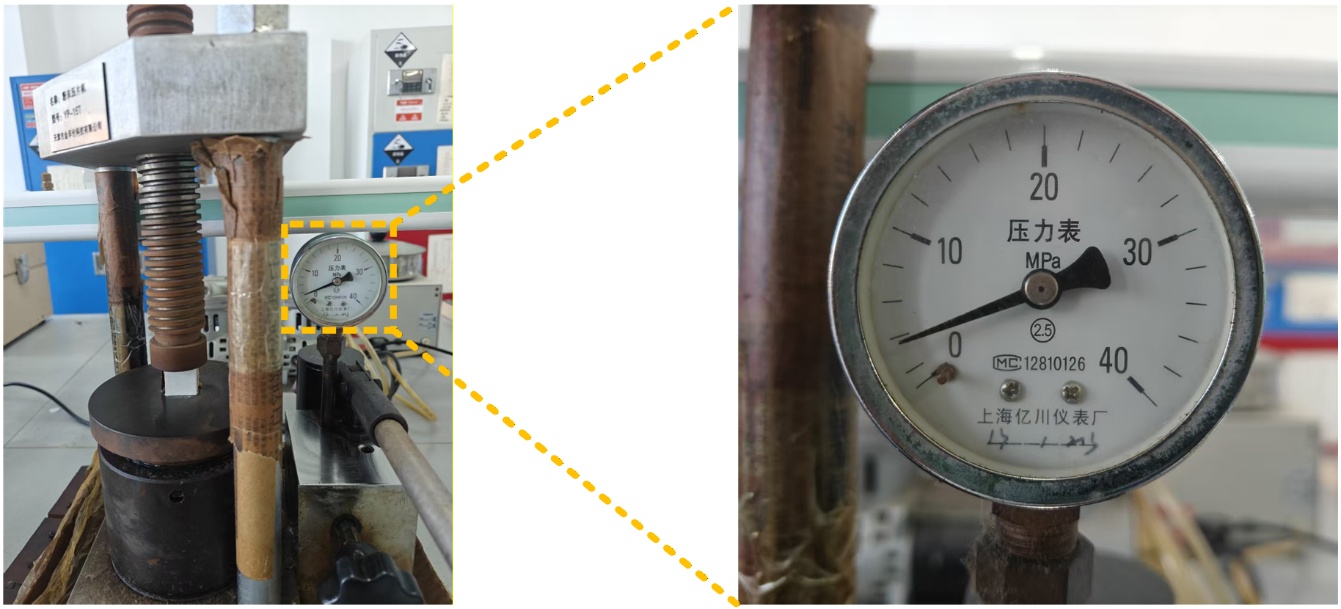
**

## Figure S3 Digital photograph of the HEG device under a pressure of 4 MPa.


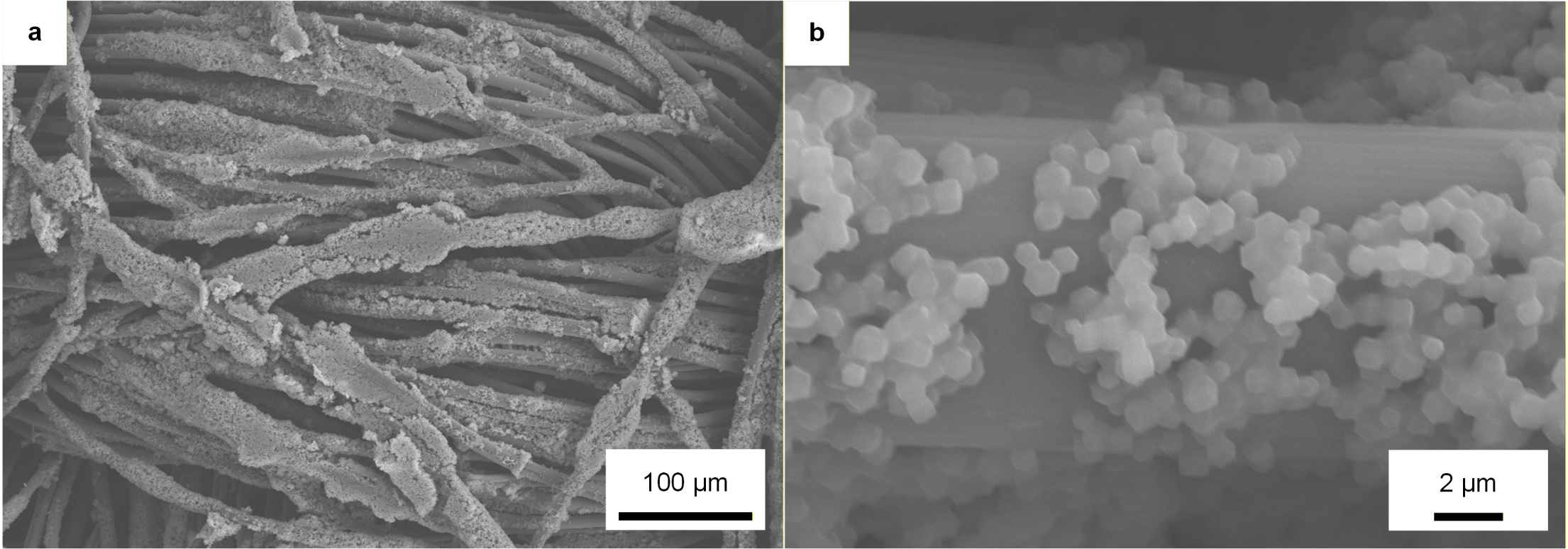


## Figure S4 SEM images of ZIF67@CC. SEM images of ZIF67@CC composite electrode at (a) low and (b) high magnifications.


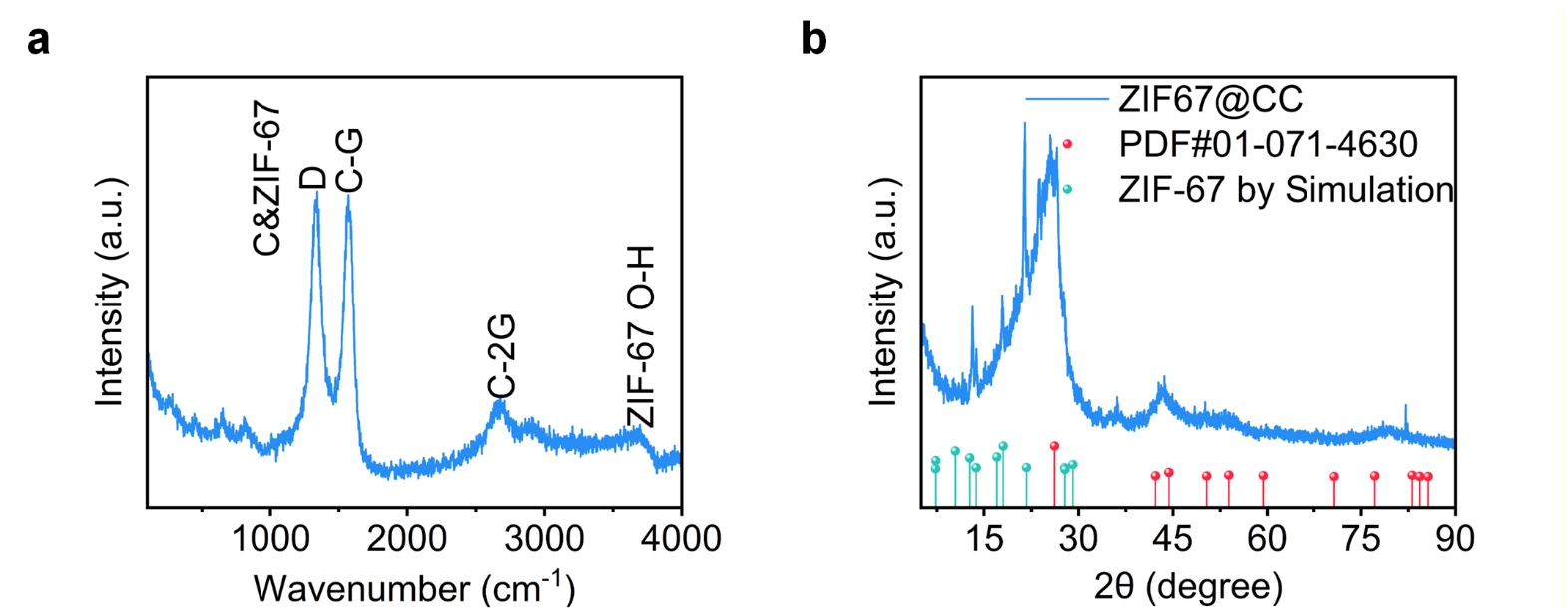


## Figure S5 Characterization of ZIF67@CC composite electrode. XRD (a) and Roman (b) spectrum of ZIF67@CC composite electrode.


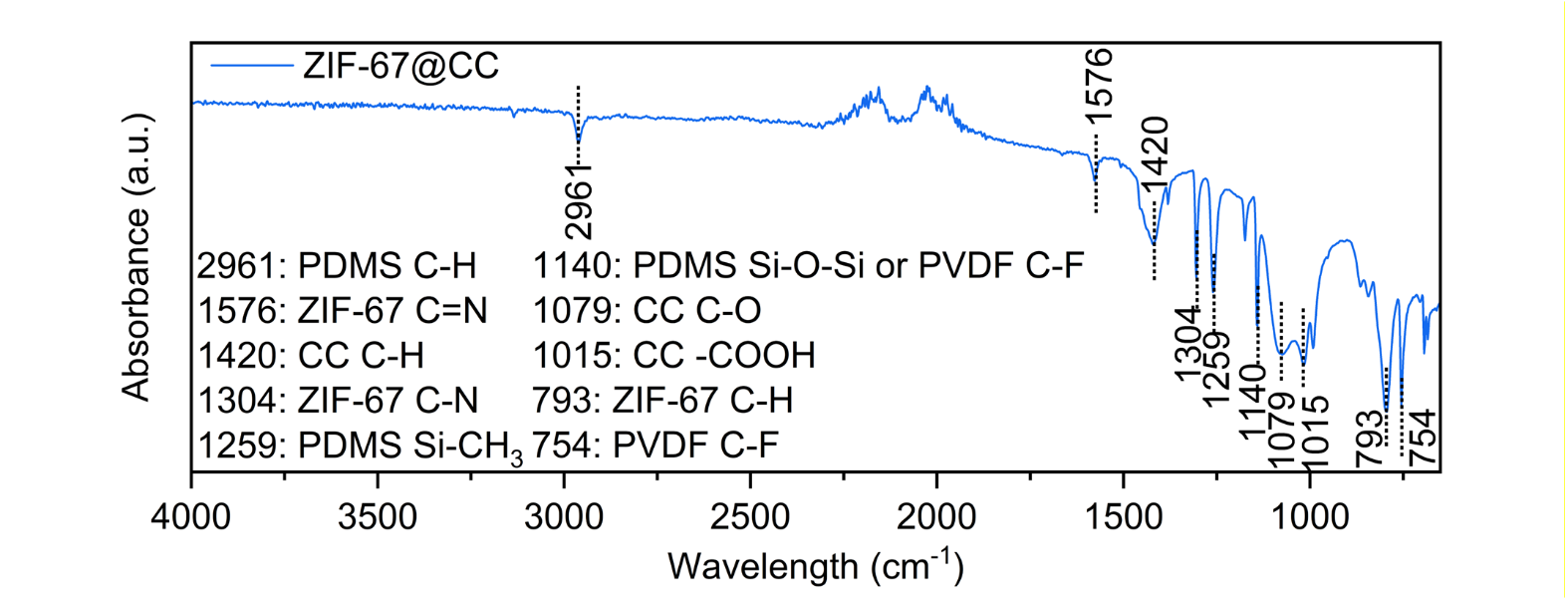


## Figure S6 Infrared Fourier transform spectra of ZIF67@CC composite electrode.


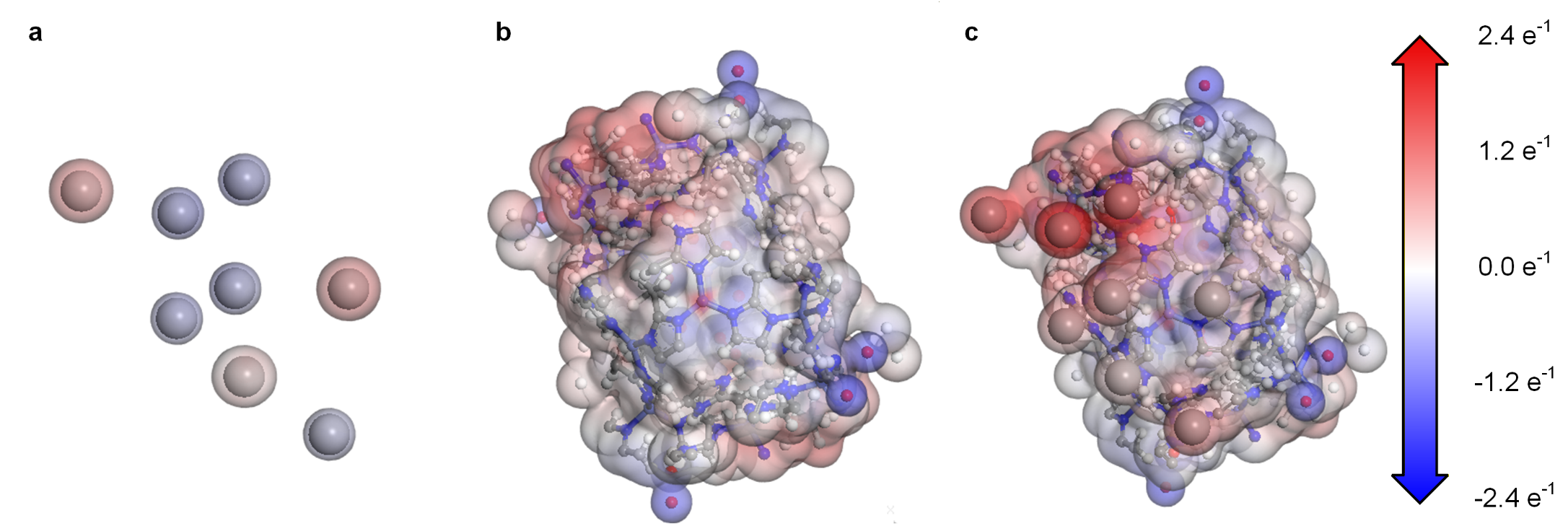


## Figure S7 Differential charge density of different electrode materials. Charge density distribution map on the surface of (a) CC, (b) ZIF-67, and (c) ZIF67@CC.


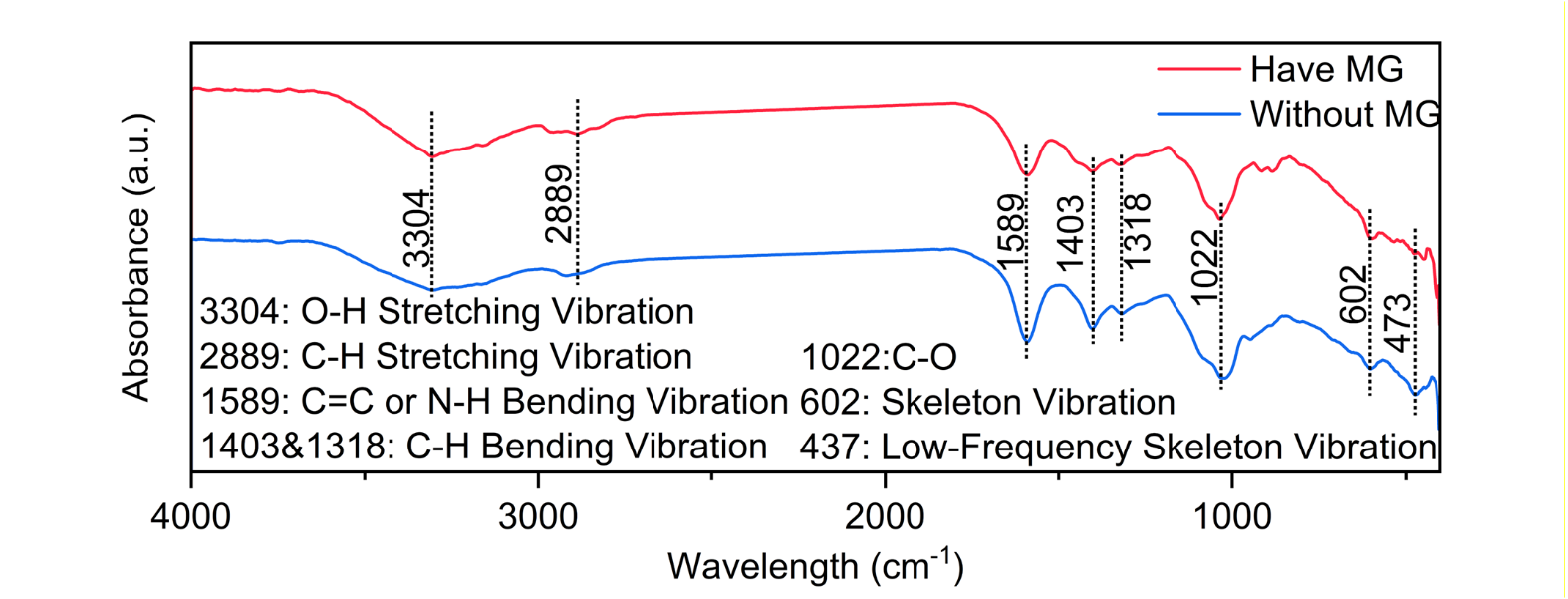


## Figure S8 Infrared Fourier transform spectra of CMC-C gel composite before and after MG.

**
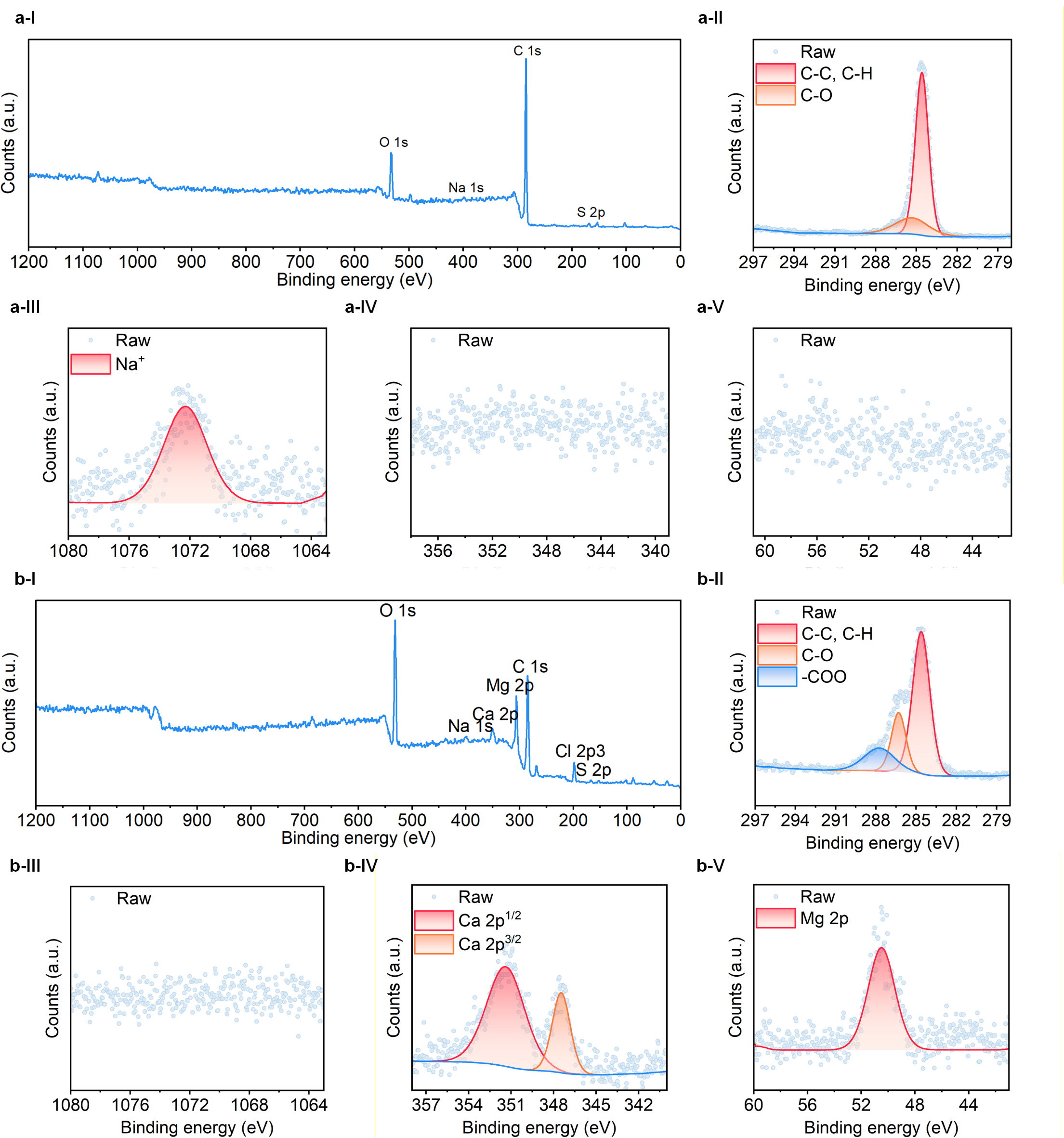
**

## Figure S9 Characterization of gel. XPS spectra of CMC-C gel composite before (a) and after (b) MG. (Ⅰ-Ⅴ) represent the following: (Ⅰ) the full spectrum, (Ⅱ) the C1s spectrum, (Ⅲ) the Na1s spectrum, (Ⅳ) the Ca2p spectrum, and (Ⅴ) the Mg2p spectrum.

Note: During the process of CMC-C gel composite with MG, Ca^2+^ and Mg^2+^ fully replace Na^+^. As Na^+^ is depleted, the -COO groups are completely exposed. This results in a significant increase in the number of oxygen-containing groups in the CMC-C composite gel, enabling the gel to absorb moisture from the air more quickly and assist in heat dissipation.


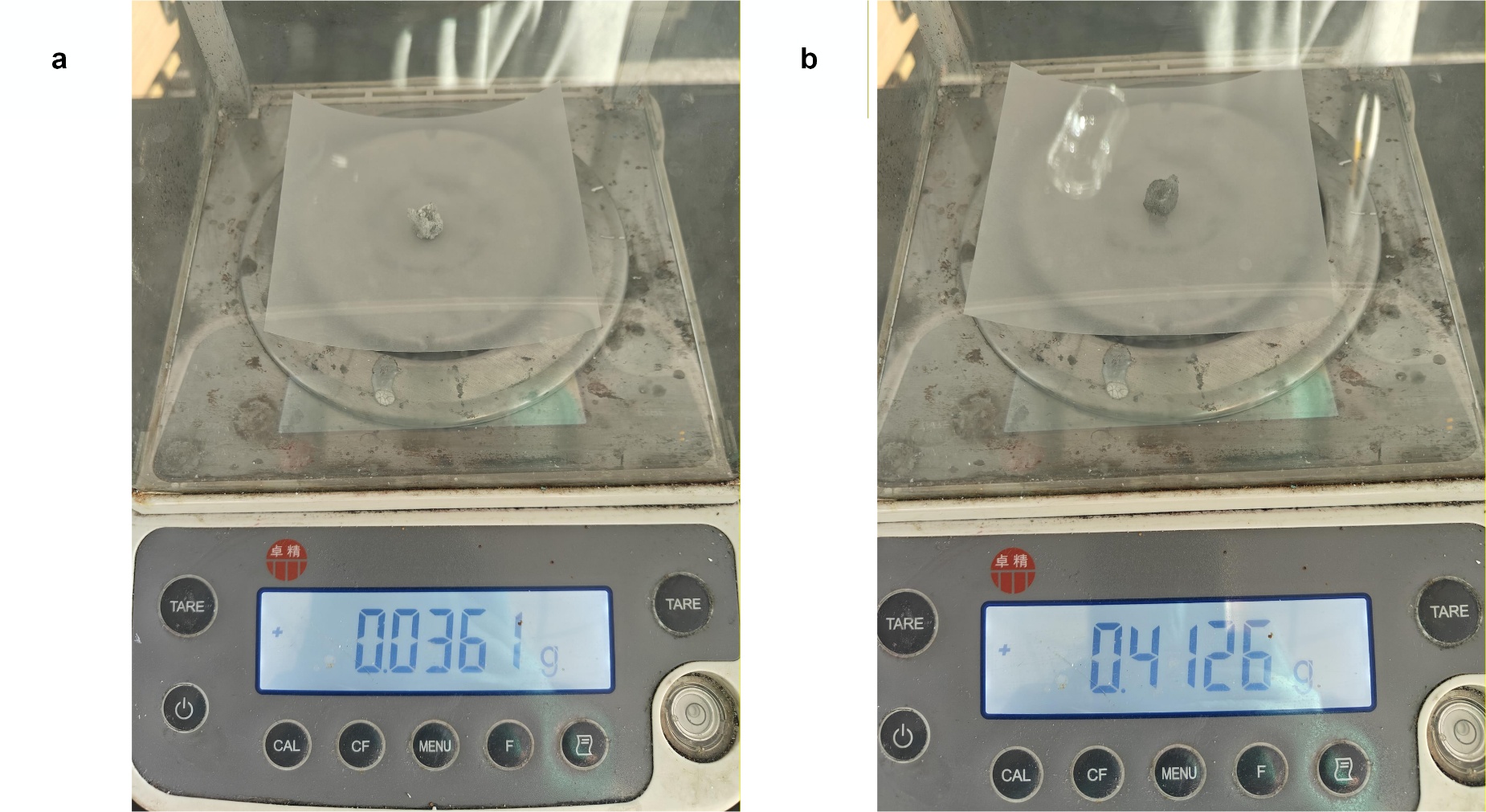


## Figure S10 CMC-C gel water absorption capacity. Mass of the (a) dried gel and the (b) gel after water absorption.


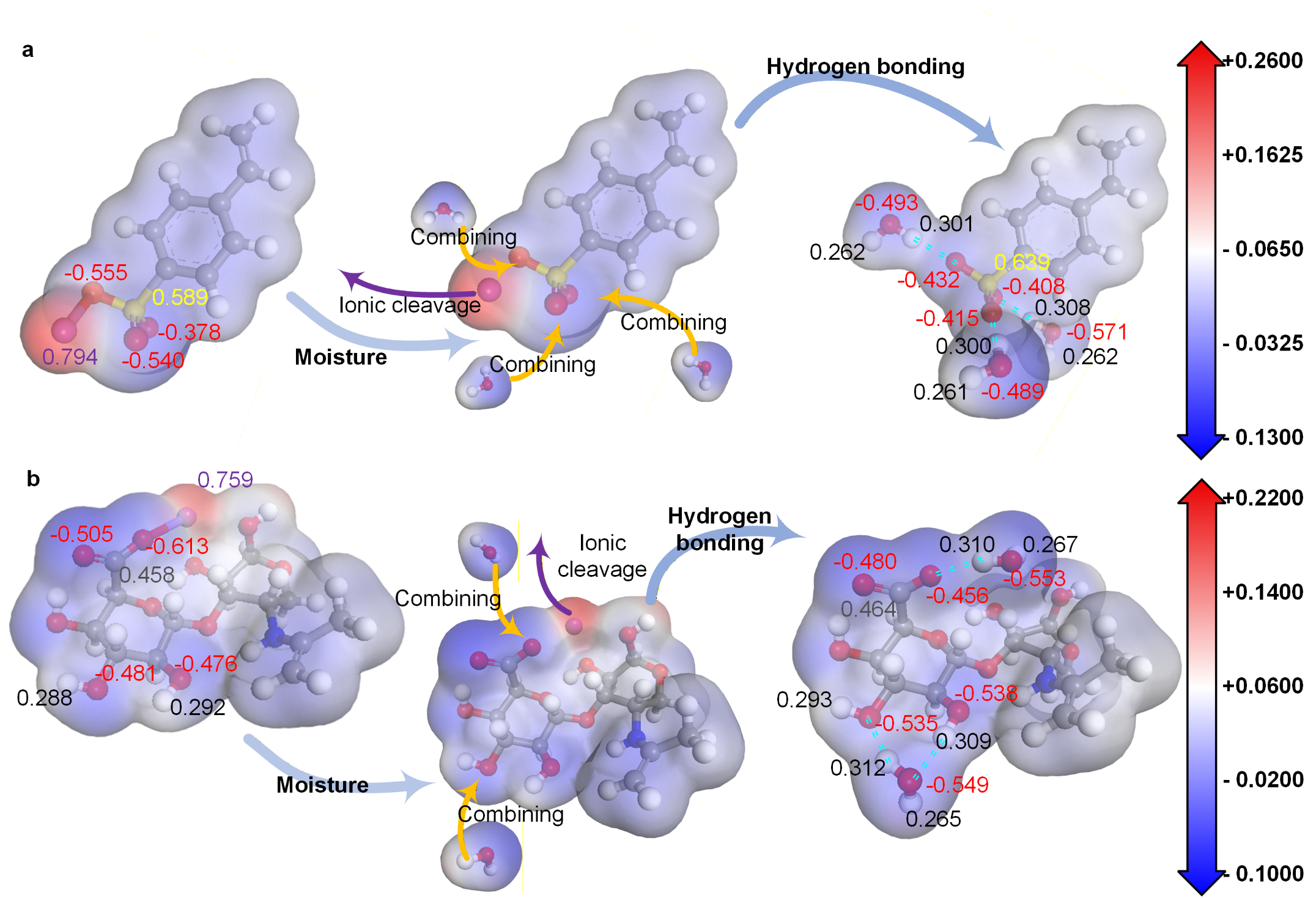


## Figure S11 Simulation of surface charge of gel monomers. The change in surface charge density of PSS (a) and sodium hyaluronate (b) before and after contact with H_2_O.


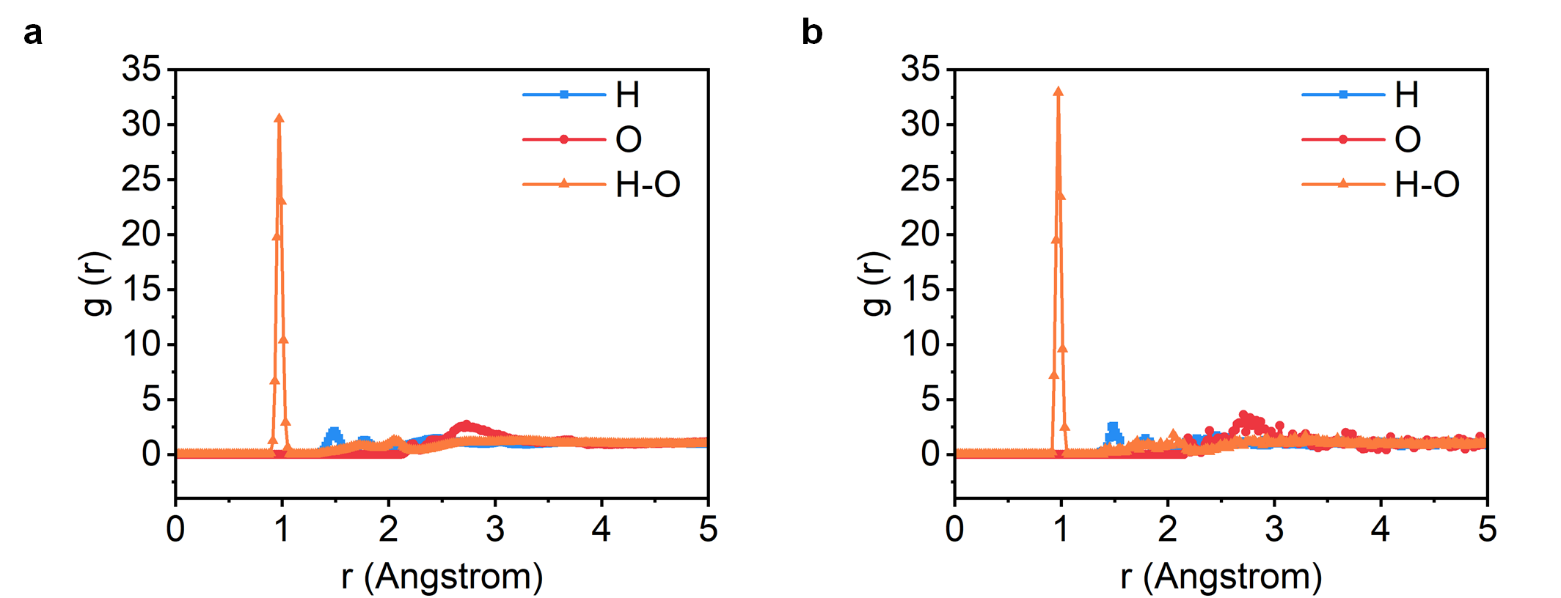


## Figure S12 The bond length distribution of O-H at different temperatures. (a) Distribution of different bond lengths at 25 ℃ and (b) at -15 ℃.


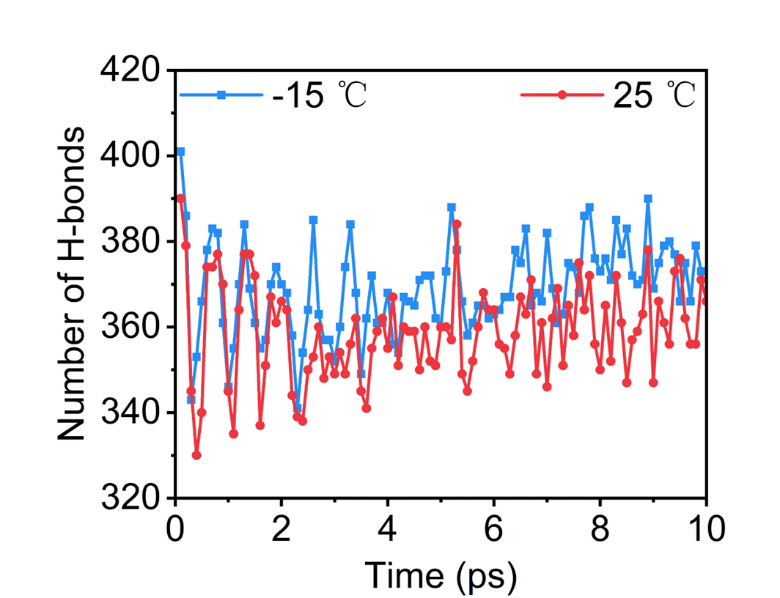


## Figure S13 The variation in the number of hydrogen bonds in the gel box at different temperatures.

Note: At different temperatures, the trend of hydrogen bond quantity variation of H_2_O in the gel is similar. At -15 ℃ (ultra-low temperature), there are only 10 more hydrogen bonds than at 25 ℃, which is negligible compared to the total number of 400 hydrogen bonds. This demonstrates that the gel effectively suppresses the transition of H_2_O from gas phase to solid phase, ensuring that the HEG can still operate at ultra-low temperatures.


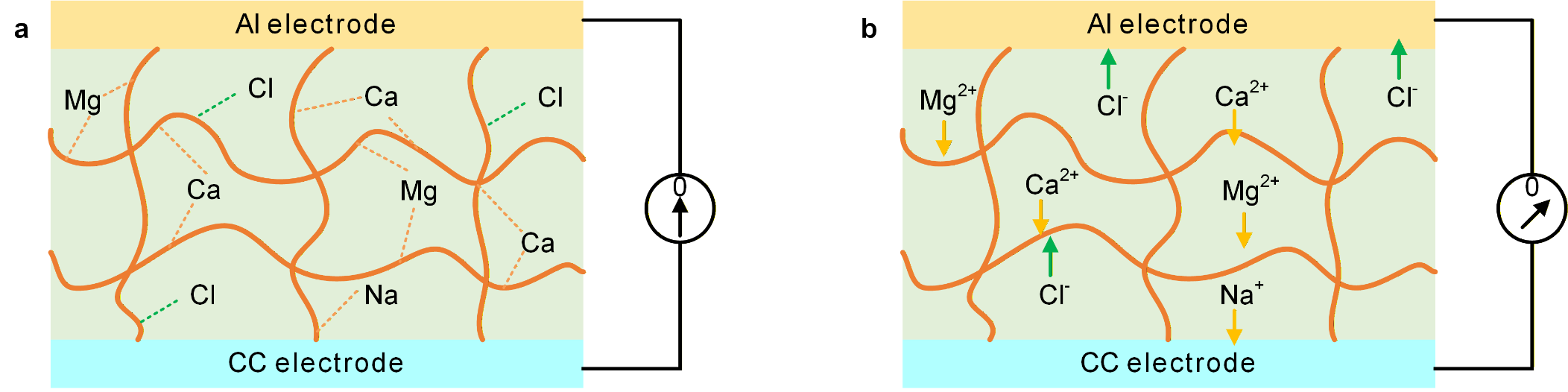


## Figure S14 Equivalent circuit diagram for load testing. Schematic diagram of the working mechanism of HEG. Schematic of ion distribution in HEG (a) under dry conditions and (b) under humid conditions. The orange arrows in panel b represent the direction of cation migration, while the green arrows indicate the direction of anion migration.

**
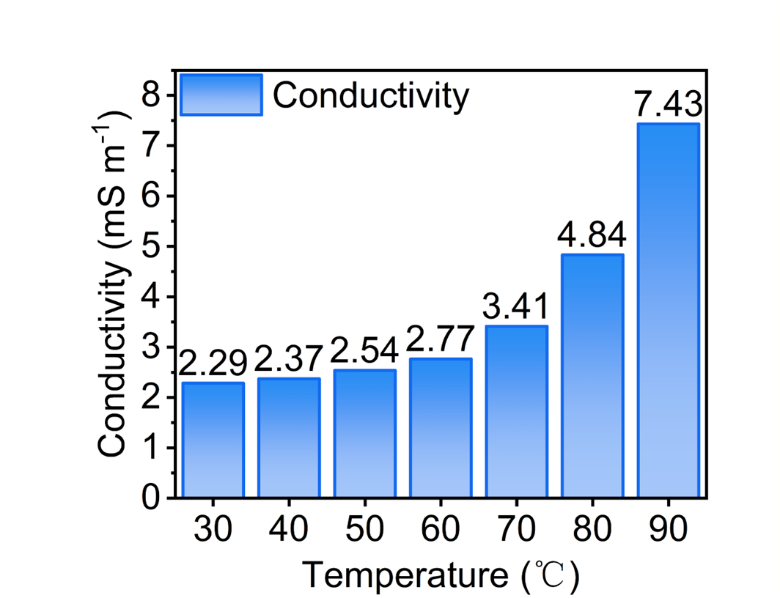
**

## Figure S15 Graph of the conductivity of cellulose-based aerogels as a function of temperature.


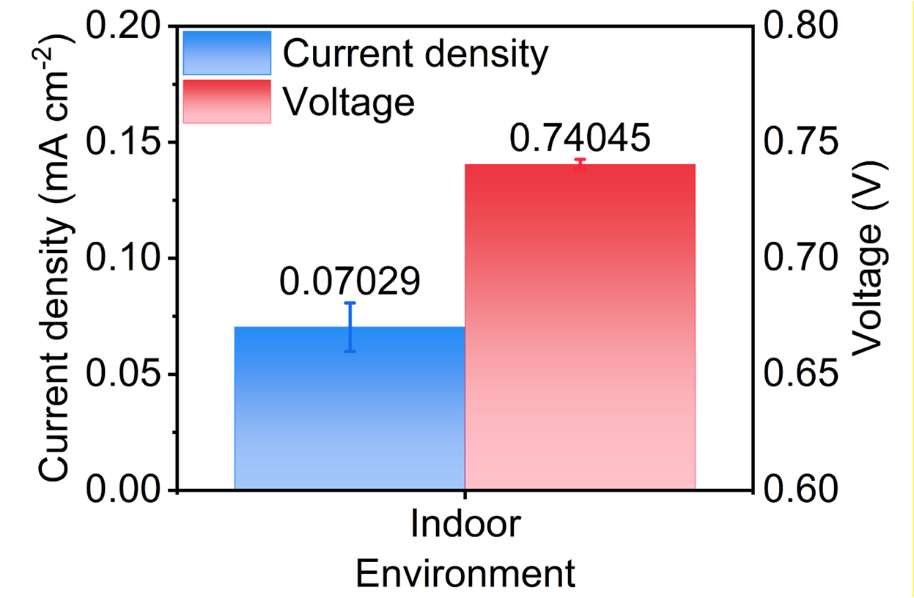


## Figure S16 Average current density and voltage of the HEG in a closed environment during the on-off state of the LED (236 nm) over a period of 30 min. Error bars represent the standard deviation.


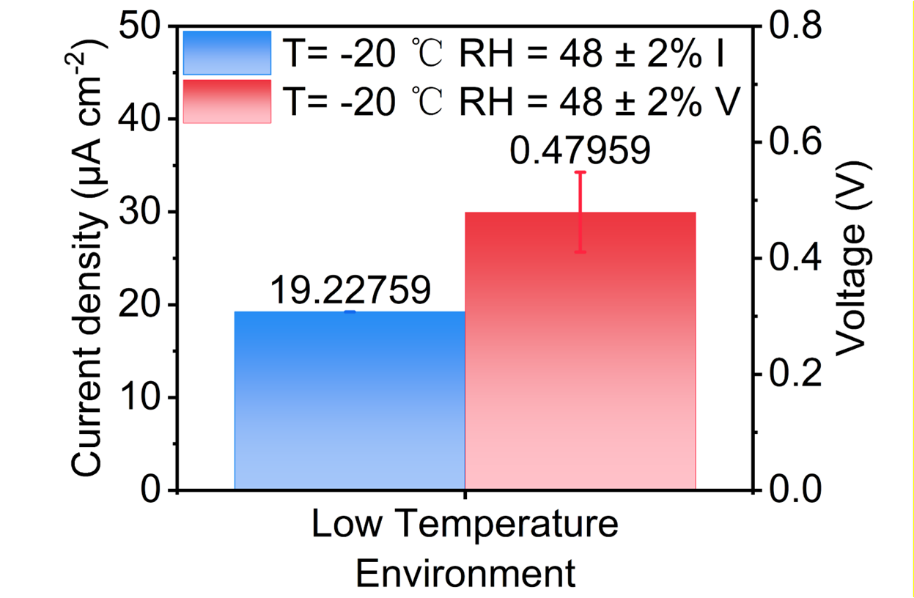


## Figure S17 The I-V curve of the HEG device over a 30 min period in a low-temperature environment. The blue line represents current density, while the red line represents voltage; the error bars indicate the standard deviation.


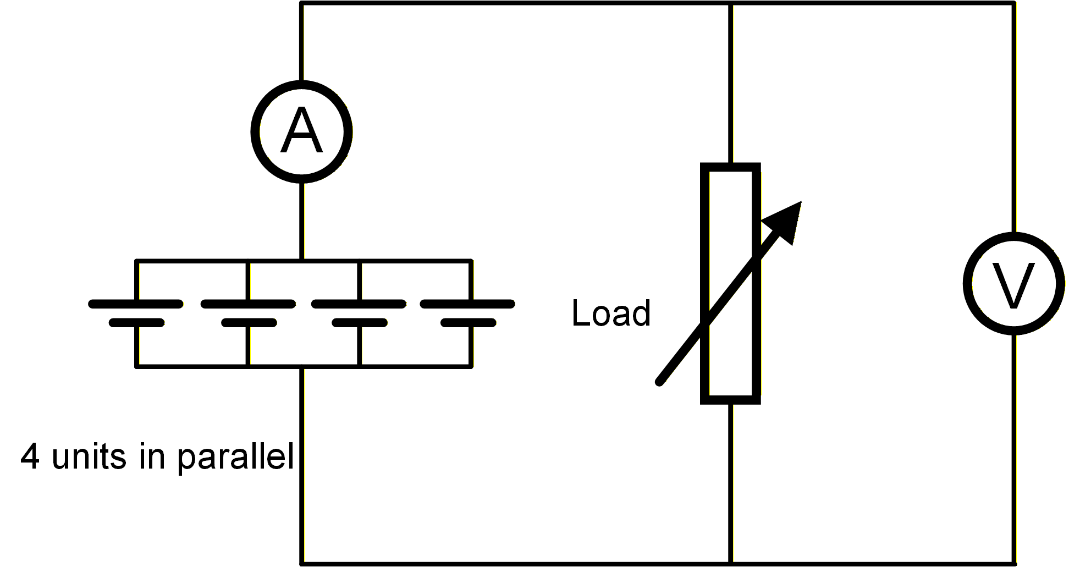


## Figure S18 Equivalent circuit diagram for load testing.

Note: Each HEG module can be regarded as composed of four small units, each with an area of 3 cm^2^.


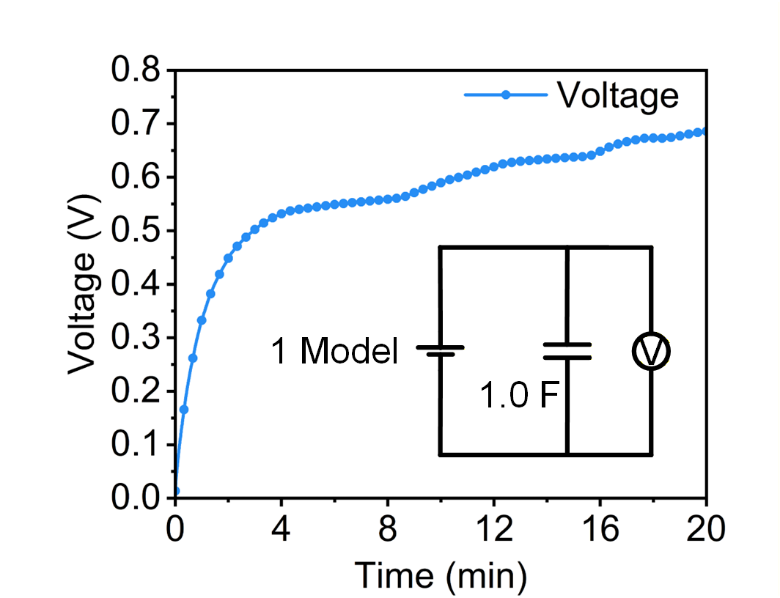


## Figure S19 Voltage variation during the charging of a 1.0 F capacitor using the LED@HEG composite device.


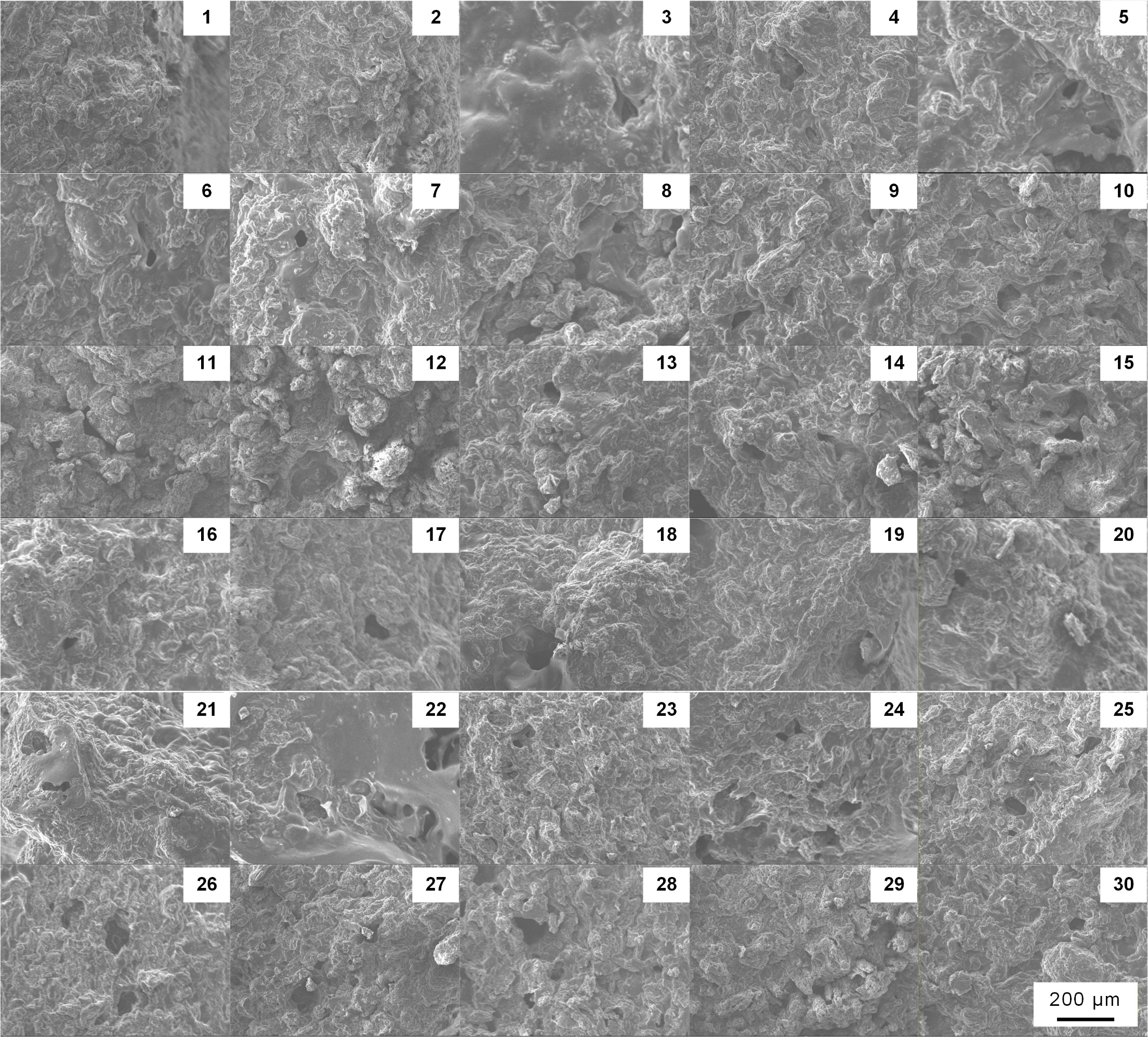


## Figure S20 30 different aerogel SEM images.


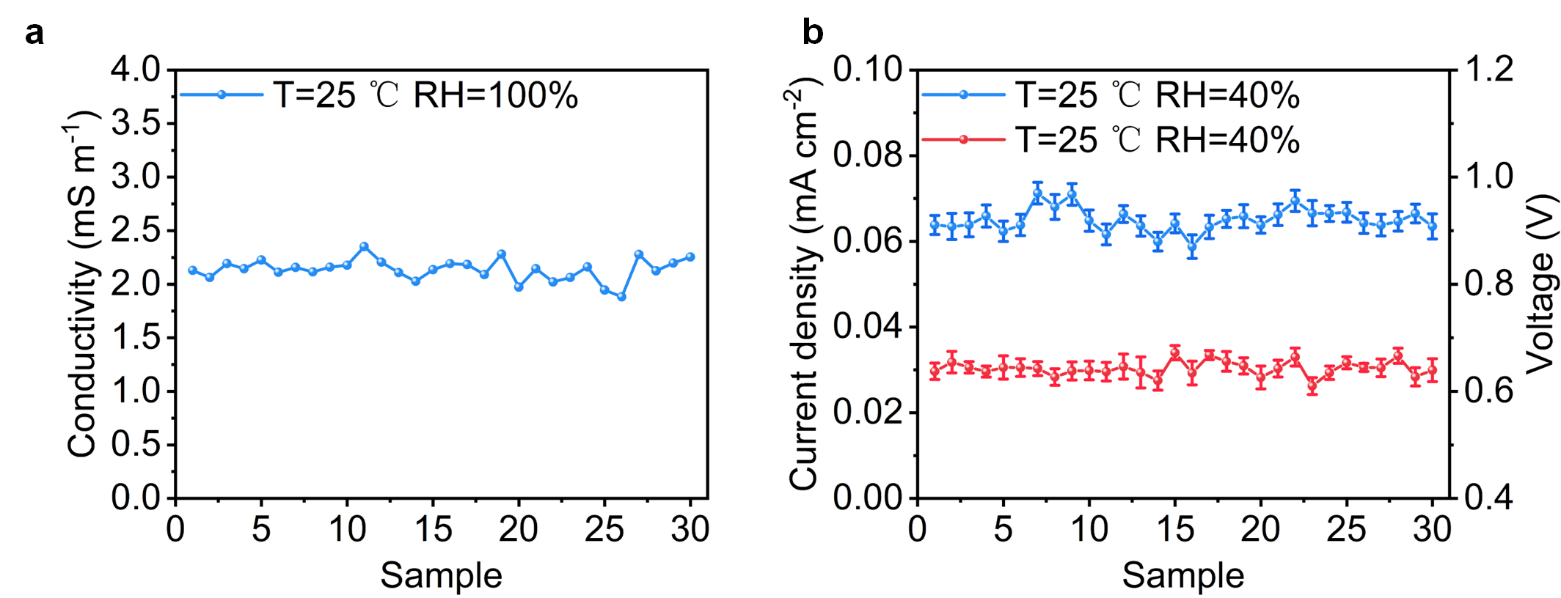


## Figure S21 Reproducibility testing of 30 HEG devices. (a) Conductivity of 30 aerogels. (b) Average I-V curves for 30 HEG devices after 30 min under conditions of 25 ℃ and RH=40%. Current density is represented in blue, and voltage is represented in red. Error bars denote the standard deviation.


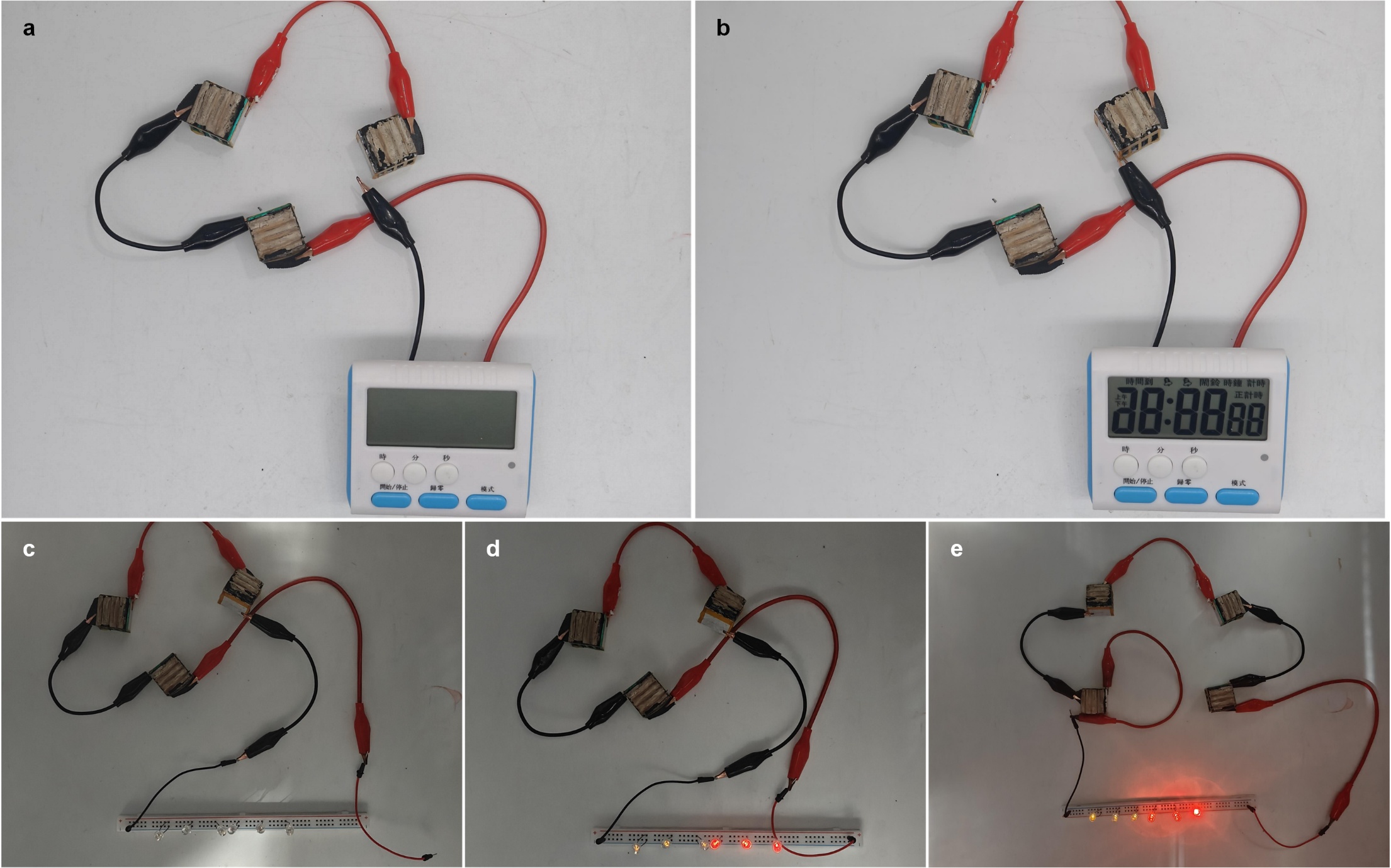


## Figure S22 Digital photos of the composite device during actual usage. (a-b) Three HEGs connected in series power the timer. (c-d) Three HEGs and (e) four HEGs connected in series power the LEDs.

Note: By connecting three HEG modules in series, they can power a timer or supply power to six parallel visible light LEDs (three yellow and three red). Moreover, as the number of series connections increases, the light intensity becomes higher.


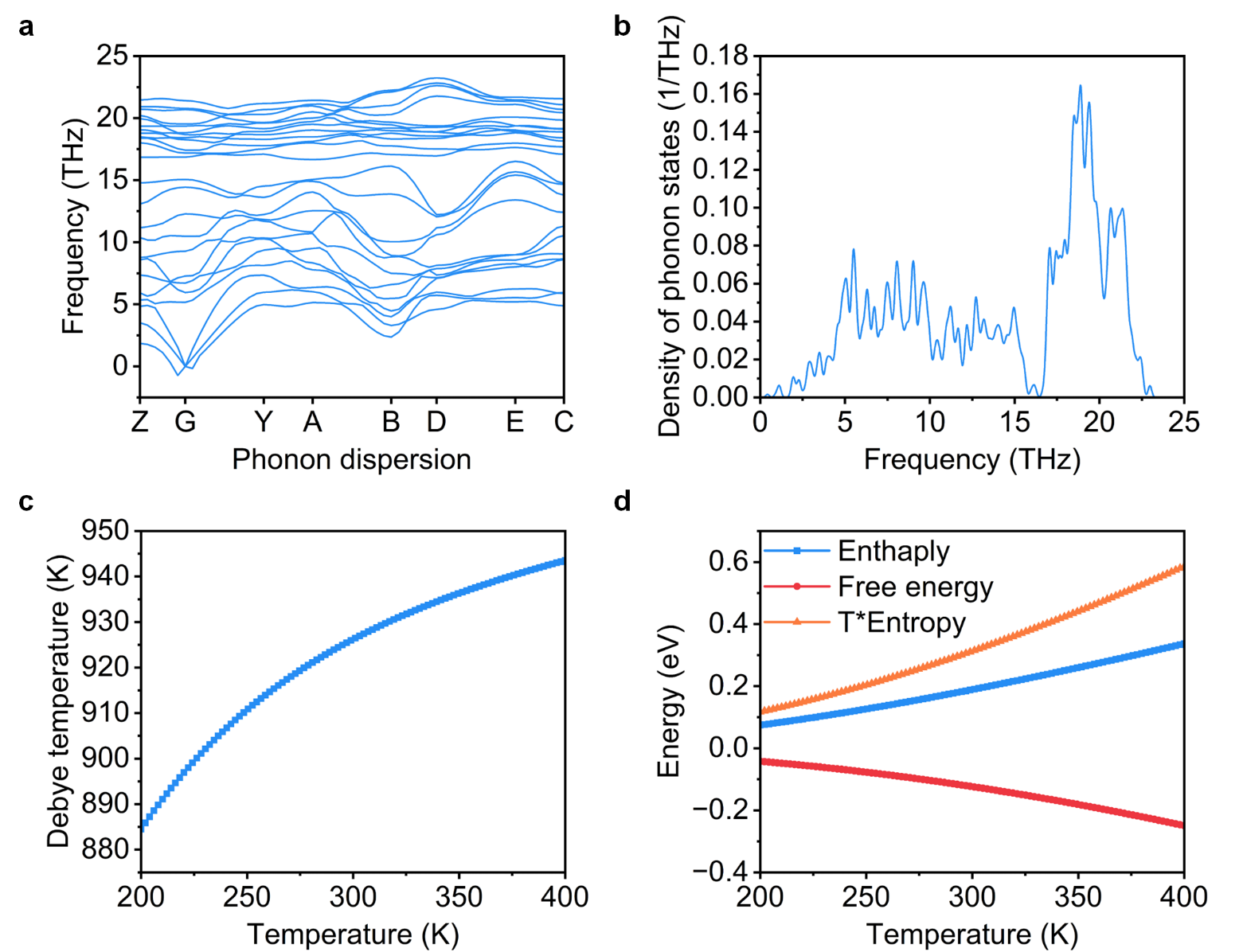


## Figure S23 First-principles-based simulation of AlGaN. (a) Phonon dispersion spectrum. (b) Phonon density of states. (c) Variation of Debye temperature over time. (d) Enthaply (blue), free energy (red), and the product of temperature and entropy (orange) as a function of temperature.


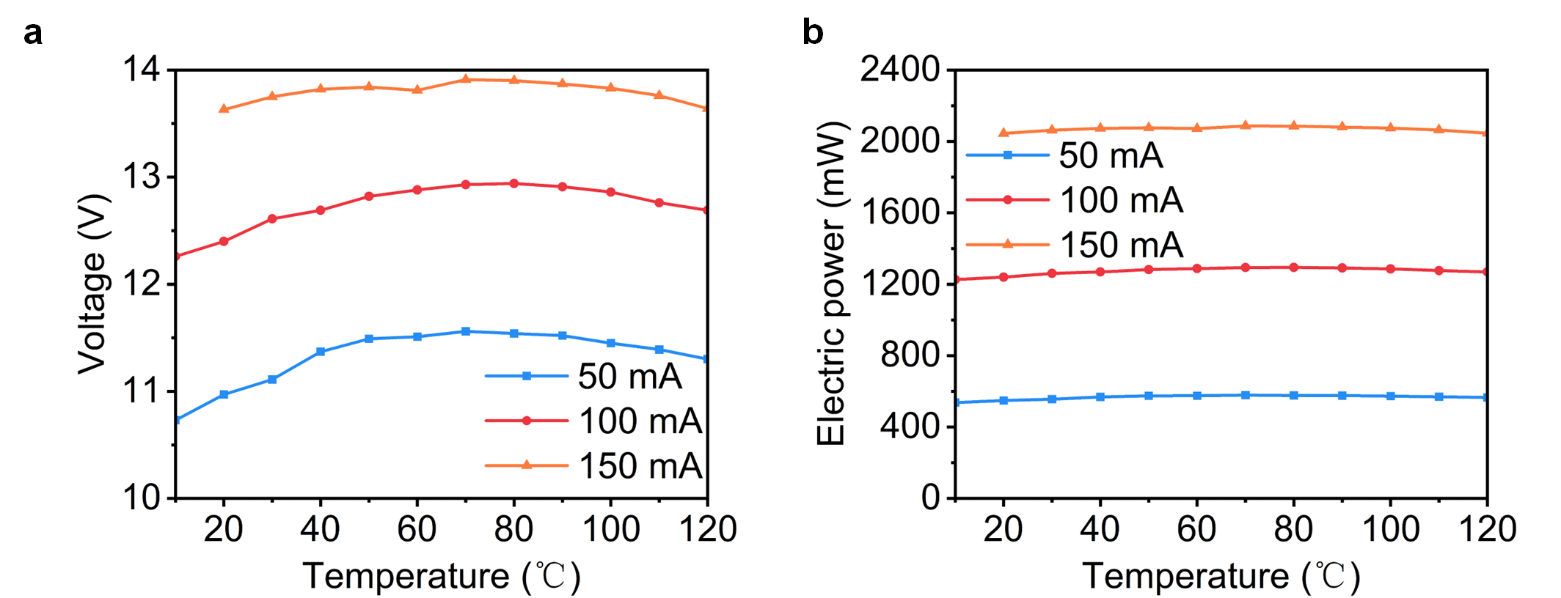


## Figure S24 LED (236 nm) electrical performance characterization. The (a) input voltage and (b) input electrical power of the LED (236 nm) under different temperatures and currents.


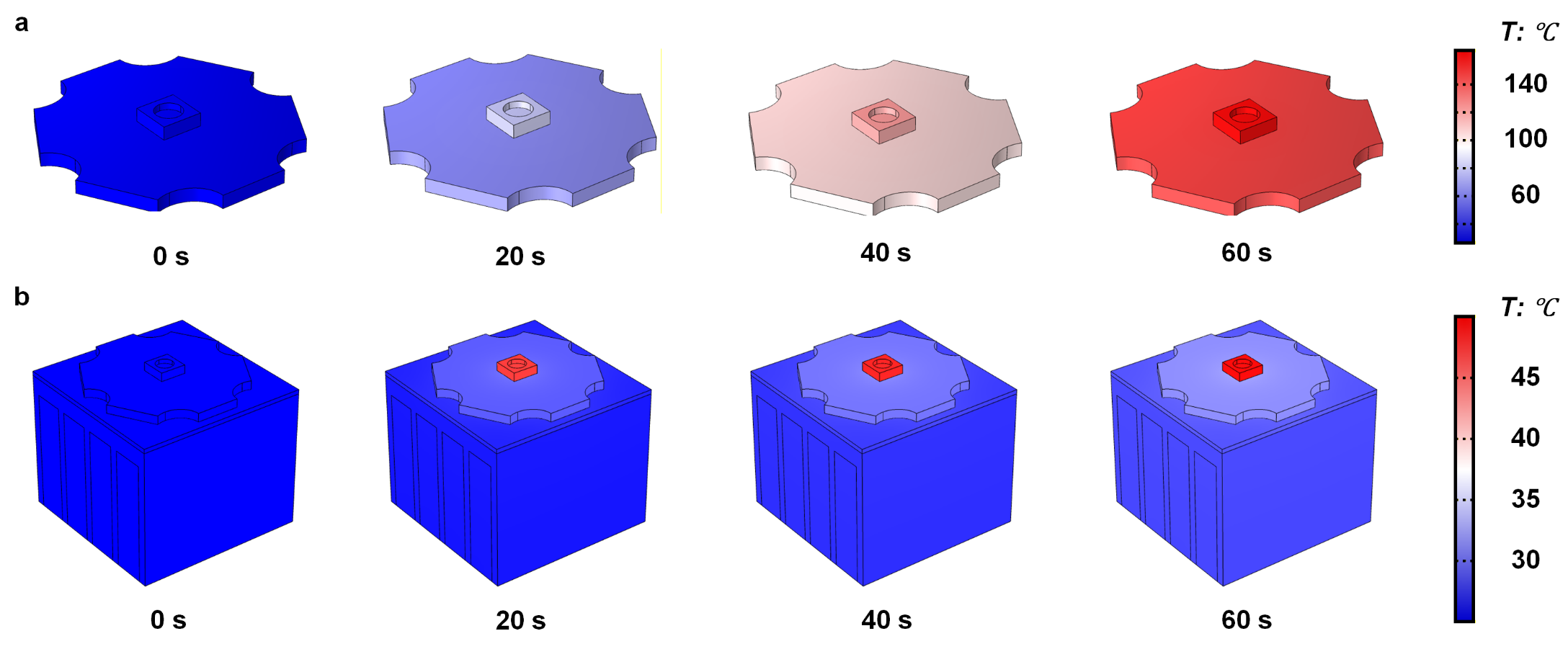


## Figure S25 Thermal finite element simulation. Finite element calculation of heat dissipation for (a) LED (236 nm) device and (b) LED(236 nm)@HEG device.


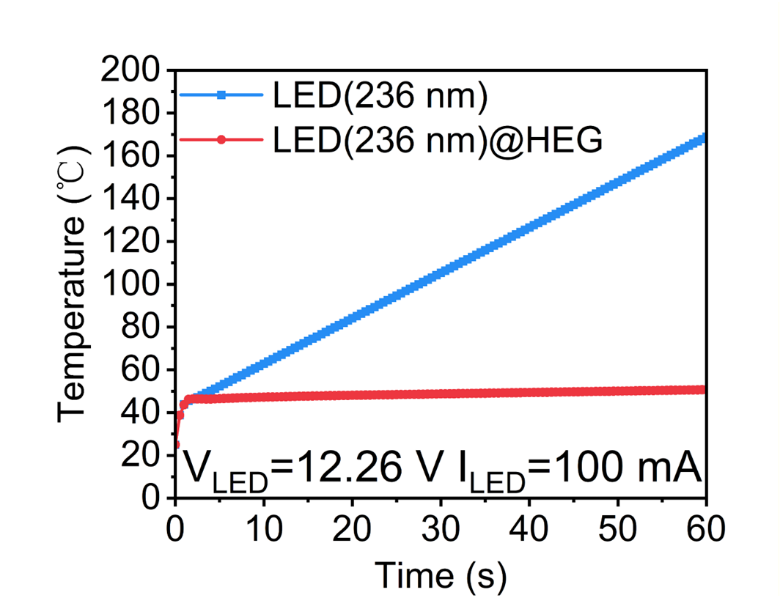


## Figure S26 Temperature variation of the LED(236 nm) before and after forming a composite device with the HEG.


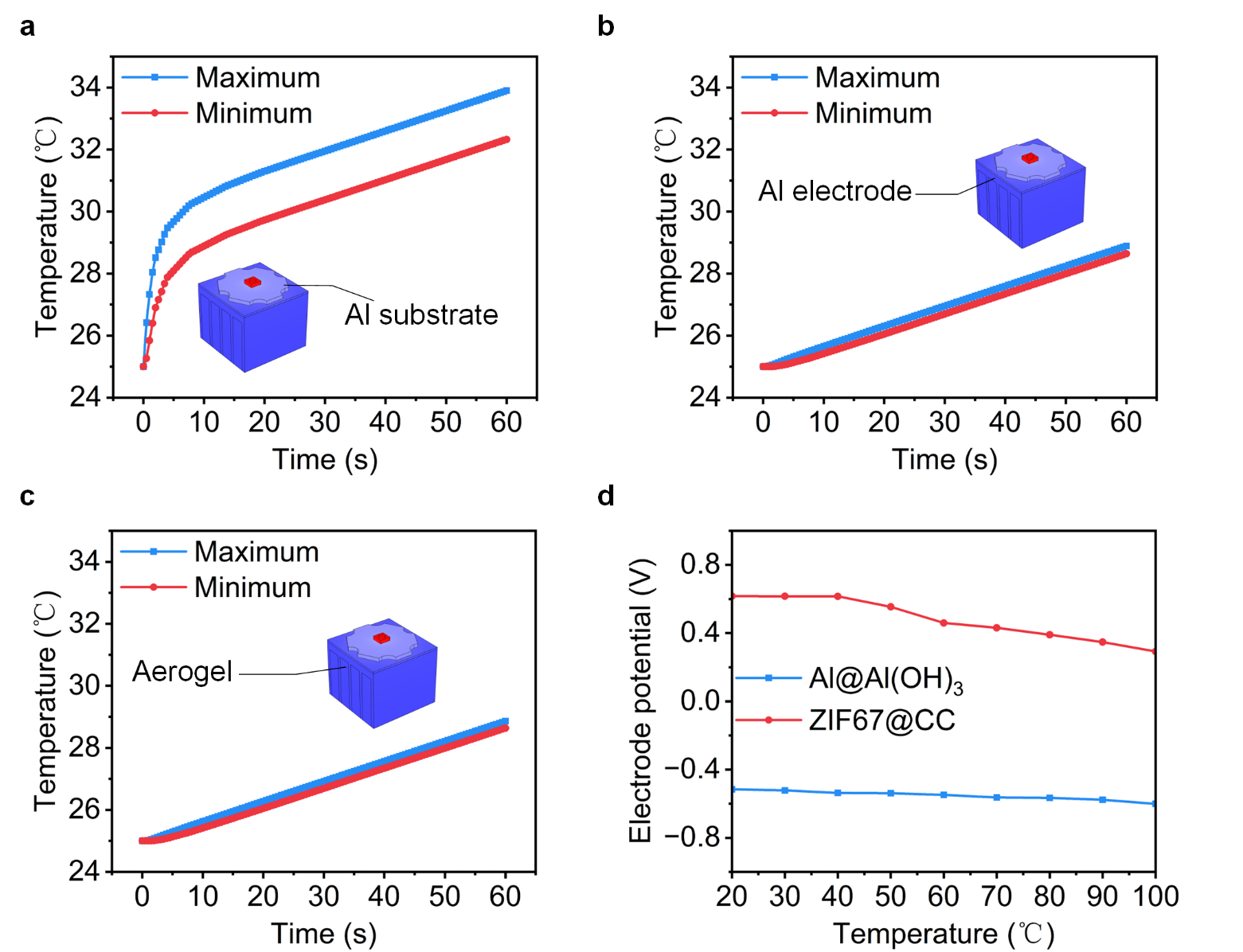


## Figure S27 Temperature finite element simulation and electrode thermal performance characterization. Variation of the maximum (blue) and minimum (red) temperatures of the (a) Al substrate, (b) Al electrode, and (c) aerogel with LED operating time. (d) Temperature-dependent electrode potential curves of Al@Al(OH)3 and ZIF67@CC composite electrodes.


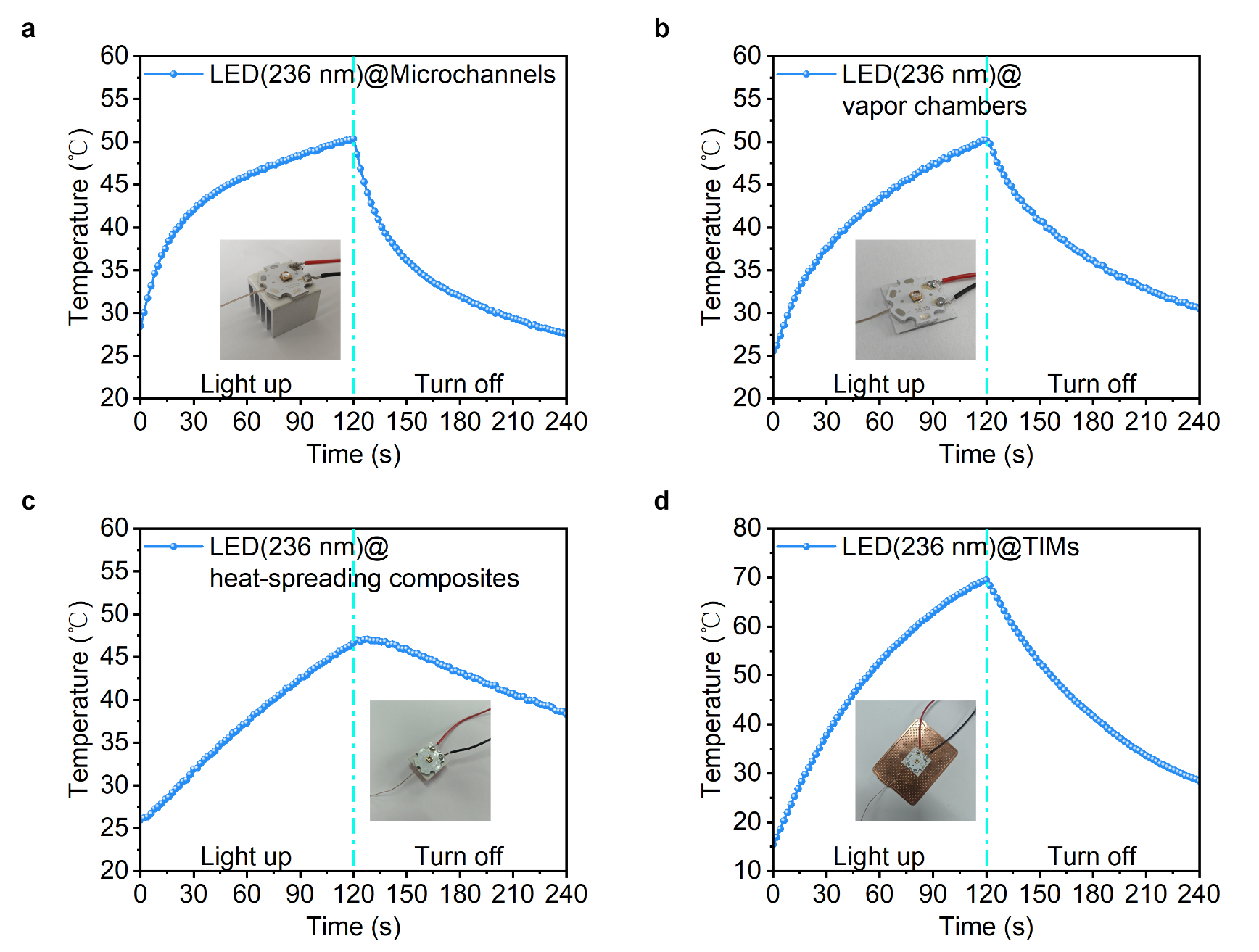


## Figure S28 The temperature variation of the LED (236 nm) with the use of passive cooling methods. The temperature changes of the LED (236 nm) under the influence of (a) microchannels, (b) TIMs, (c) heat-spreading composites, and (d) vapor chambers are presented. The illustrations in the figure are digital photographs taken while using different cooling methods.


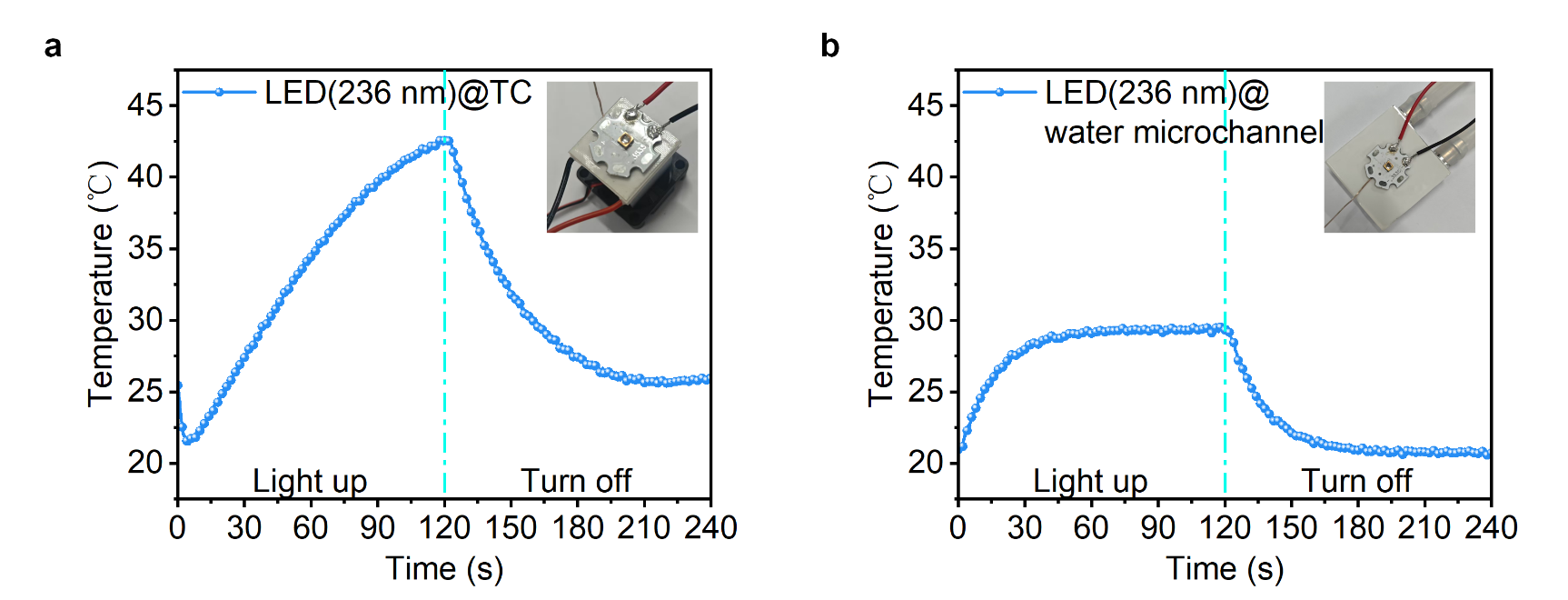


## Figure S29 The temperature control of the LED (236 nm) using active cooling methods. The temperature changes of the LED (236 nm) under the effects of (a) water microchannels and (b) thermoelectric cooling are depicted during a 2 min operation followed by a 2 min pause

**
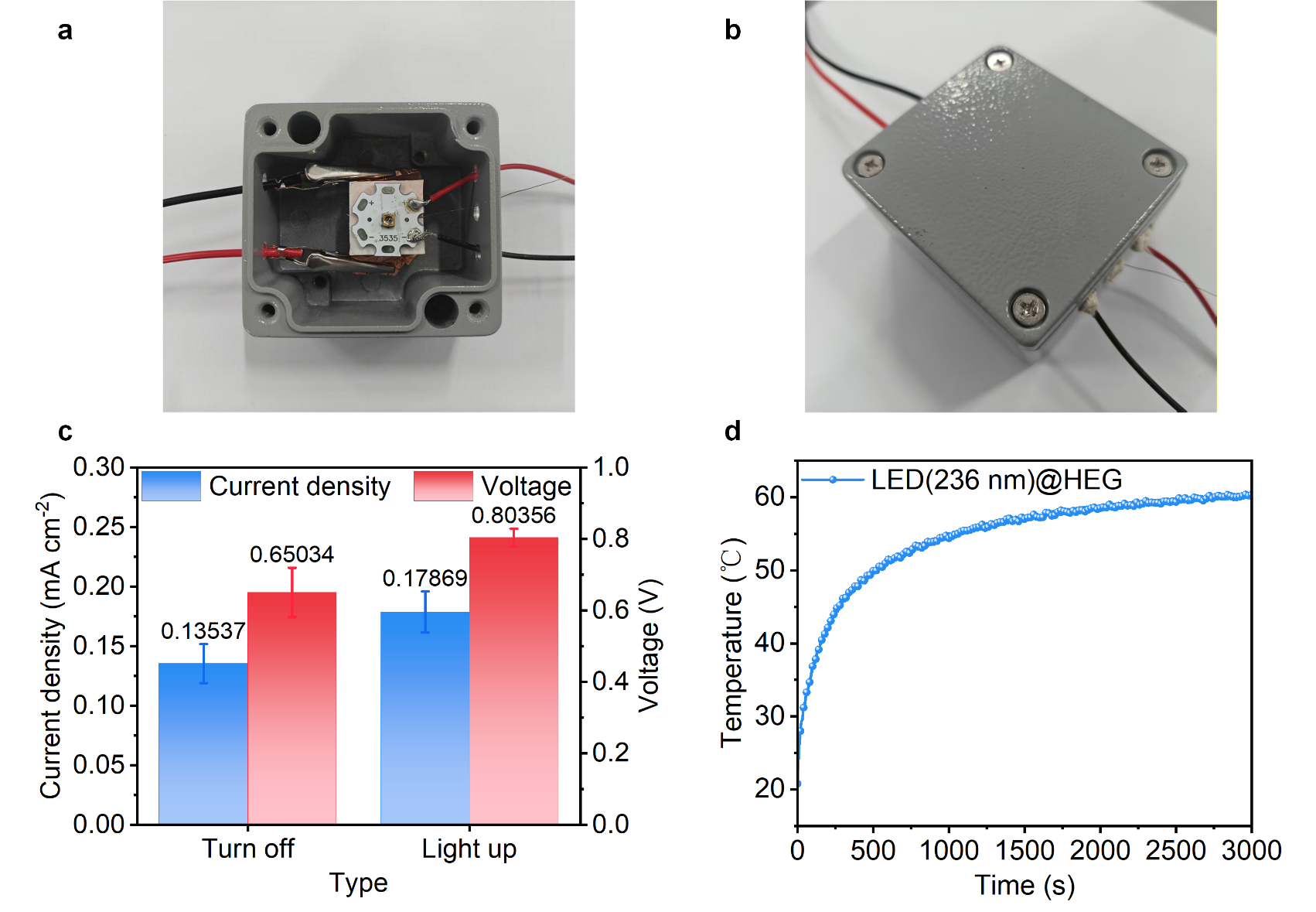
**

## Figure S30 Performance testing of the LED (236 nm)@HEG composite device in a closed environment. Digital photographs of the sealed box: (a) interior and (b) exterior. (c) Average current density and voltage of the HEG in a closed environment during the on-off state of the LED (236 nm) over a period of 30 min. Error bars represent the standard deviation. (d) Temperature variations of the LED (236 nm)@HEG when supplied with 100 mA and 12.26 V of electrical power in a closed environment.


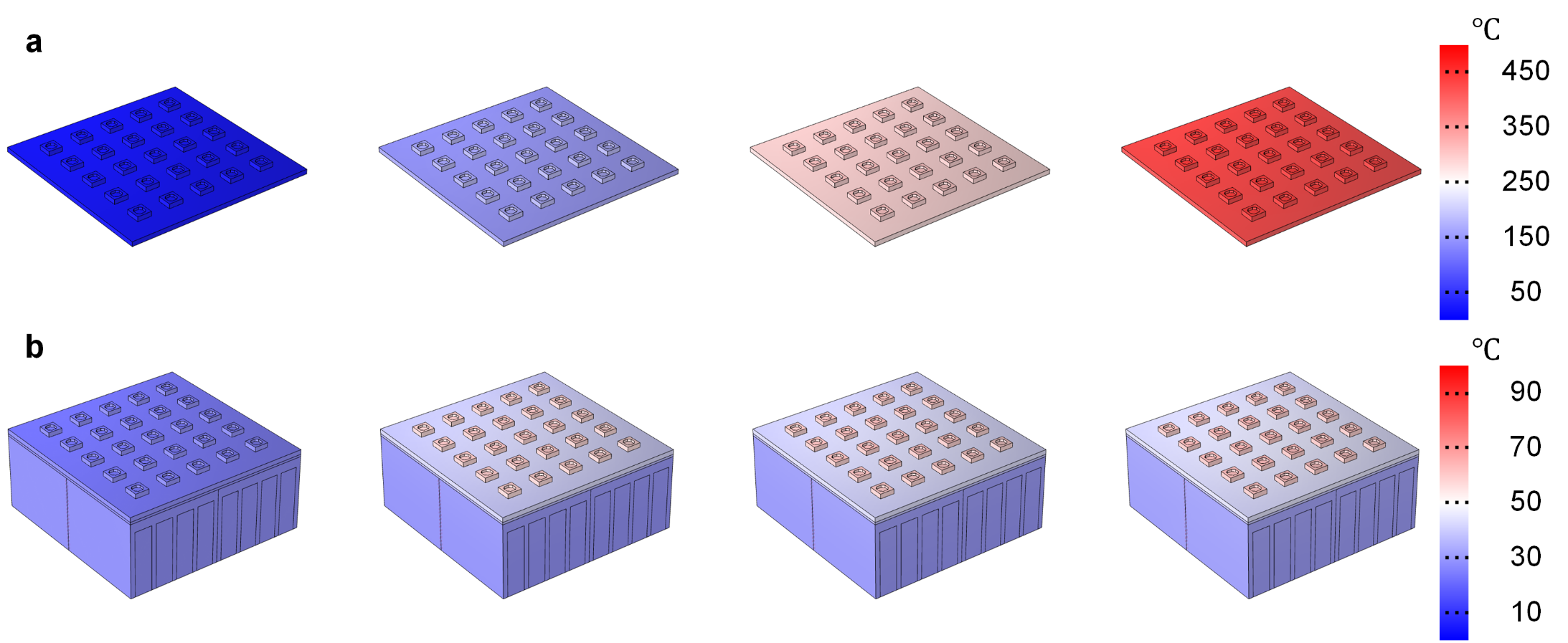


## Figure S31 Finite element analysis of large integrated devices. Finite element calculation of heat dissipation for 25LED(236 nm) device (a) and 25LED(236 nm)@HEG device (b).


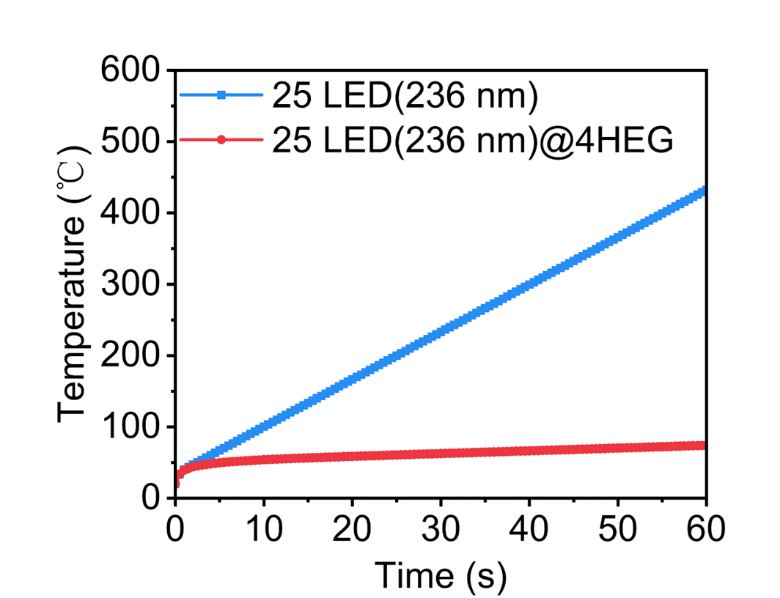


## Figure S32 Temperature variation of the 25 LED(236 nm) before and after forming a composite device with the 4 HEG.

**
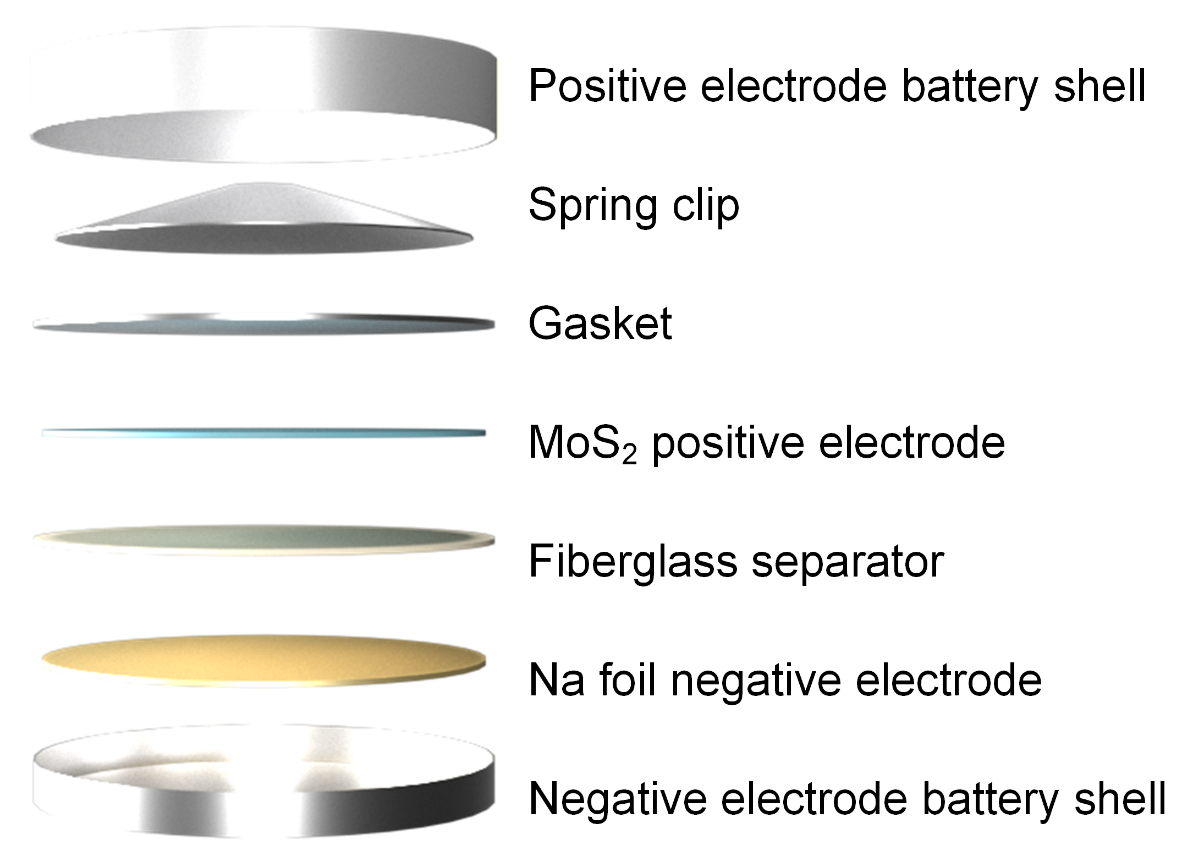
**

## Figure S33 Schematic diagram of the sodium-ion battery structure.


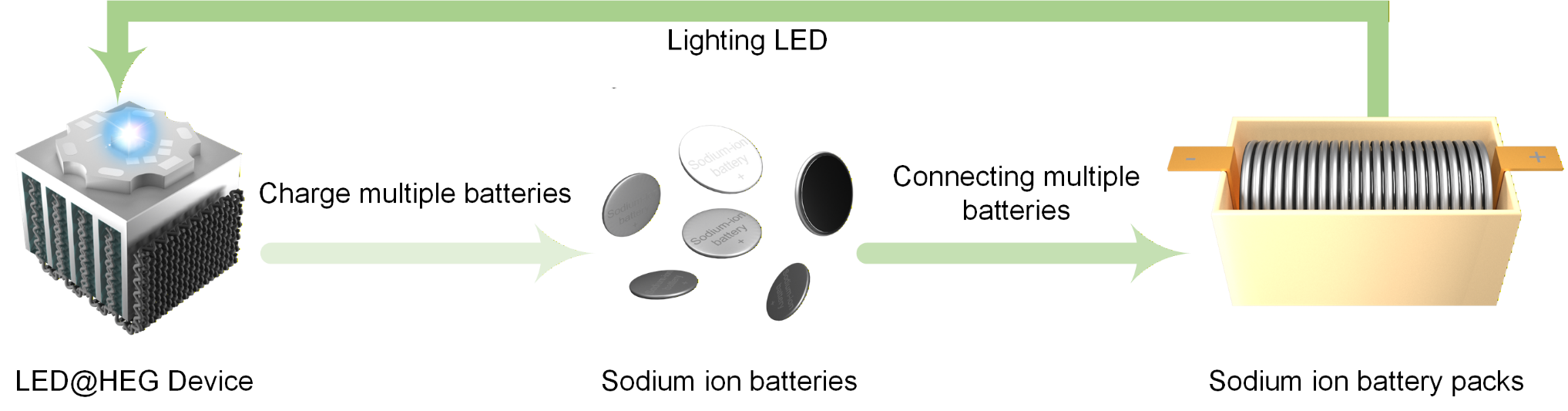


## Figure S34 Schematic diagram of energy cycling.


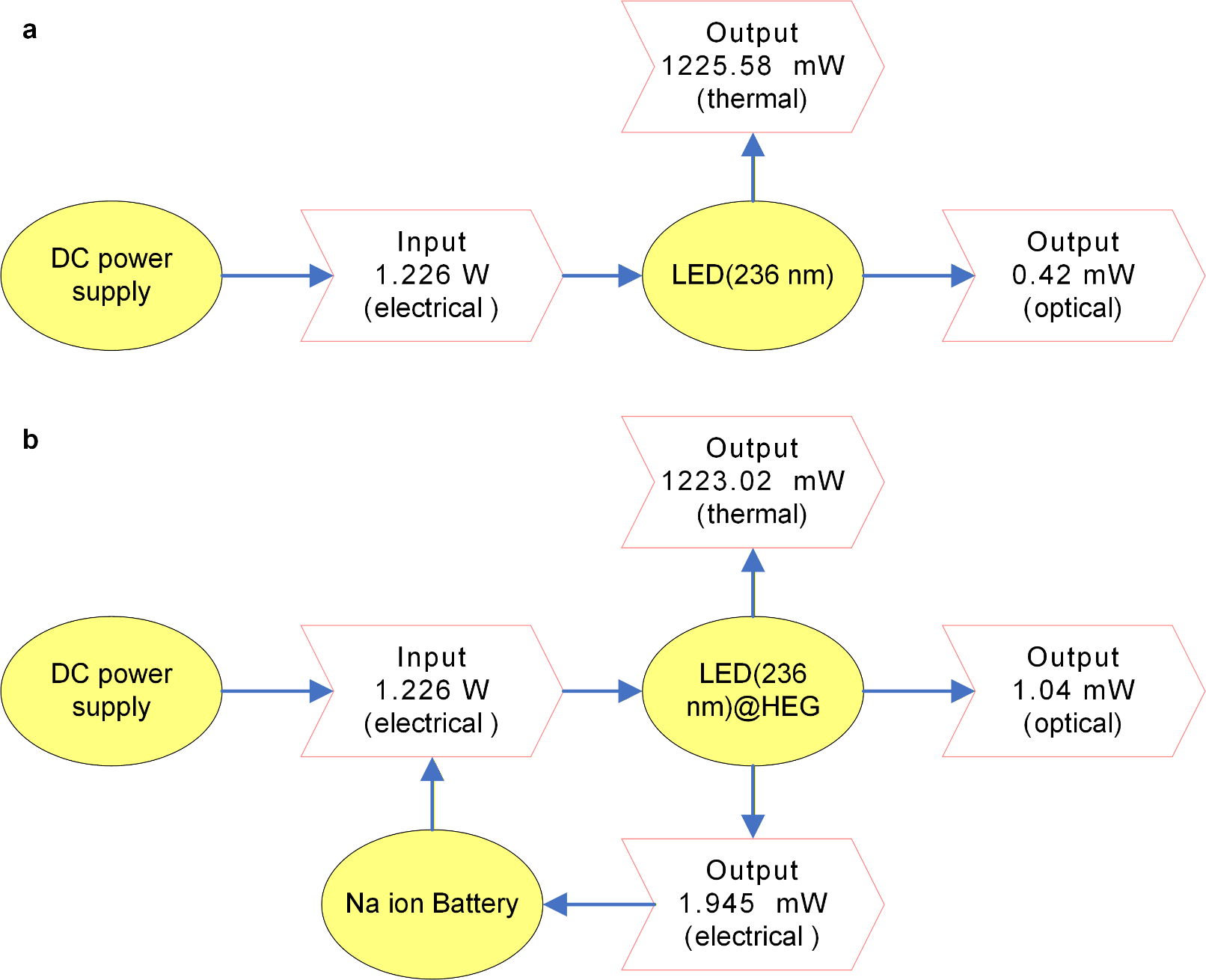


## Figure S35 Energy flow diagrams. (a) LED(236 nm) and (b) LED(236 nm)@HEG energy flow diagrams.


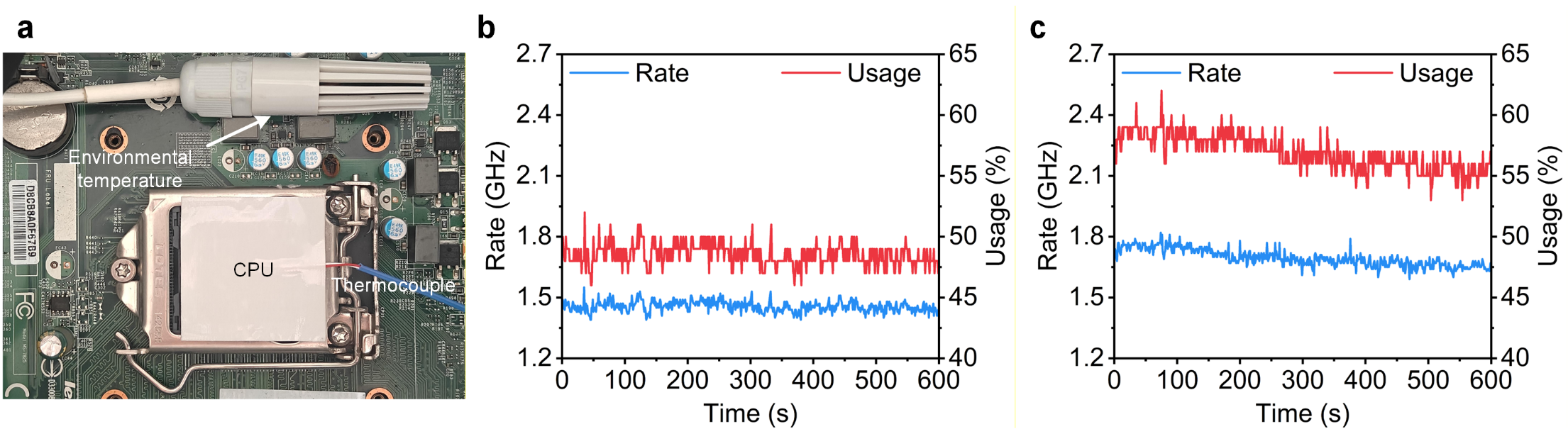


## Figure S36 Application of HEG on the Intel G3220 chip. (a) Digital photo of the motherboard equipped with the G3220 chip. The temperature probe is positioned on the surface of the chip (blue line), and the environmental measurement is on the side of the chip (white cylinder). Variation curves of the chip's processing rate (blue) and usage (red) (b) with and (c) without HEG deployment.


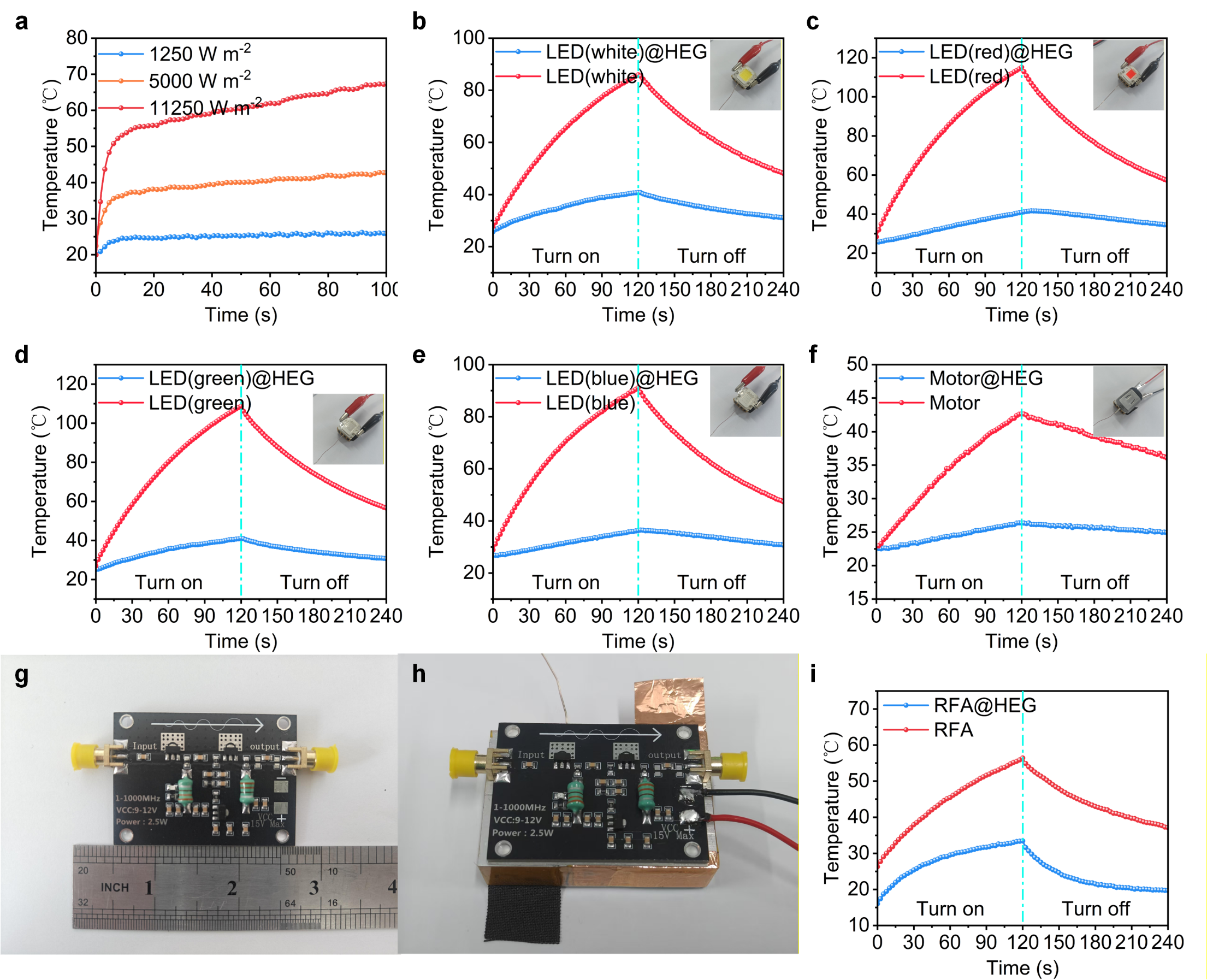


## Figure S37 HEG Cooling Performance Testing. (a) Temperature variations of HEG under different heat flux densities. Temperature variations of high-power (b) white light, (c) red light, (d) green light, and (e) blue light LEDs before and after integration with HEG. (f) Temperature changes of the motor before and after integration with HEG. The inset in the figure is a digital photograph of the heating device after integration with HEG. (g) Digital photos of the RFA module and (h) RFA@HEG device. (i) Variation of RFA core temperature with usage time before and after using HEG.

# Supplementary Table

## Table S1 The binding energy of different components of the composite gel with H_2_O.

|  | E_A+H2O_  Ha | E_A_  Ha | E_H2O_  Ha | E_H2O-A_  Ha |
| --- | --- | --- | --- | --- |
| CMC-C | -1362.020266 | -1592.984216 | -229.134450 | 460.0984 |
| SH | -1505.462650 | -1514.995083 | -152.756599 | 162.289032 |
| SA | -2356.570995 | -2127.397378 | -229.134536 | -0.03908 |
| PSS | -1161.455367 | -932.280615 | -229.134450 | -0.040302 |

## Table S2 Comparison of device output current, voltage, and optimal power.^1-12^

| Ref. | Year | Electrodes | Mesosphere | Current density  (μA cm^-2^) | Voltage  (V) | Power density  (μW cm^-2^) | Environment |
| --- | --- | --- | --- | --- | --- | --- | --- |
| S1 | 2025 | Carbon conductive tape with an aluminum | SA/PVA/CNT-COOH/AETA | 23.8 | 1.03 | 9.12 | T=25 ℃  RH=60% |
| S2 | 2025 | Zn-C | SA/aANFs | 2.52 | 1.25 | 0.78 | RH=80% |
| S3 | 2025 | Ag-Cu | PAMS/AAm/LiCl | 173 | 0.89 | - | RH=60% |
| S4 | 2024 | CC-CC | g-O-BP@PVA | 0.16 | 0.221 | 0.0104 | RH=80% |
| S5 | 2024 | Cu-Cu | VO-PSS/NI-AC-Cl | 5.26 | 0.61 | 1.06 | RH=90% |
| S6 | 2024 | Zn-C | PSS-PVA | 76.41 | 0.36 | 11.24 | RH=99% |
| S7 | 2025 | Cu-Cu | Mn^Ⅱ^-Fe^Ⅲ^@CM-PSS | 60 | 0.48 | - | One sun |
| S8 | 2025 | Graphite electrodes | cellulose-based filter paper/PAM hydrogel | 12.5 | 0.6 | 1.61 | - |
| S9 | 2024 | Cu-Cu | Carbon foam | 14.4 | 0.33 | - | One sun |
| S10 | 2024 | Conductive carbon paste-Cu mesh | DBW/CBW | 148 | 0.77 | 2.07 | 1.2 M  CaCl_2_ in Water |
| S11 | 2024 | Cu-Pt | SCNF/PVA | 92 | 0.9 | 24 | RH=80% |
| S12 | 2023 | CG-rGO@TEEG | - | 590.1 | 0.44 | 17 | 4 M NaCl in Water |
| This Work | 2025 | ZIF67@CC-Al | CMC-C | 620 | 0.81 | 93.14 | Without LED chip  T=25±1 ℃  RH=95±5% |
|  |  | ZIF67@CC-Al | CMC-C | 1254 | 0.75 | 162.08 | Have LED chip  T=25±1 ℃  RH=95±5% |

Note: Sodium alginate (SA), Poly(vinyl alcohol) (PVA), Surface-modified carbon nanotubes (CNT-COOH), 2-(acryloyloxy)-ethyl] trimethylammonium chloride (AETA), Activated aramid nanofibers (aANFs), 2-Acrylamide-2-methylpropanesulfonic acid (AMPS), Acrylamide (AAm), Carbon cloth (CC), Black phosphorous (BP), the 2D V_2_O_5_ flakes with Poly(4-styrenesulfonic acid) (VO-PSS), Rectangular charcoal mask (CM), Adhesive polyacrylamide (PAM), Delignified balsa wood (DBW), Cellulosic balsa wood (CBW), sulfated cellulose nanofibers (SCNF).

## Table S3 Comparison of the Effects of Different Cooling Methods

|  | Heat Flux  (W m^-2^) | ΔT  (℃) | Electrical Power  (W) | Response Time  (s) | Footprint  (mm) |
| --- | --- | --- | --- | --- | --- |
| Water Microchannels | 4824 | 8.2 | 3.0 | 13.2 | 55 × 40 × 12  (Additional 10 L cold water basin) |
| Thermoelectric Cooling | 4824 | 17.17 | 3.3 | 143 | 30 × 30 × 31 |
| Microchannel | 4824 | 21.69 | - | 162 | 20 × 20 × 16 |
| TIMs | 4824 | 53.86 | - | 198 | 20 × 20 × 1 |
| Heat-spreading Composites | 4824 | 20.62 | - | 282 | 20 × 20 × 4.5 |
| Vapor Chambers | 4824 | 24.61 | - | 253 | 68 × 50 × 2 |
| **HEG** | **4824** | **8.32** | **-** | **232** | **20 × 20 × 16** |

Note: Here, the heat flux is 4824 W m^-2^, and ΔT represents the temperature change of the LED (236 nm) after 2 min of operation. The electrical power indicates the energy consumed by the cooling device during operation, the response time refers to the duration required for the LED (236 nm) to return to room temperature after operating for 2 min, and the footprint represents the physical dimensions of the cooling apparatus.

# Supplementary Video

## Video S1 Temperature variation of the LED(236 nm) module, LED(236 nm)@HEG device.

Note: Based on finite element simulations of the temperature changes on the surface and inside the LED during operation, comparing the temperature variations of the LED and LED@HEG composite device reveals that HEG effectively mitigates the overheating of the LED.

## Video S2 LED(236 nm)@HEG driving a small fan.

Note: This video demonstrates that by using a 1.0 F capacitor as the energy storage device for HEG, the composite device is capable of driving a small fan after charging for a period of time. This proves that the LED@HEG composite device can achieve multi-stage thermal management for the LED in practical applications without requiring additional external energy.

## Video S3 LED(236 nm)@HEG driving a LED.

Note: Using a self-made sodium-ion battery as the energy storage device for HEG, this video solves the problem of discharge platform decline in capacitors during use, achieving a stable charge-discharge platform. It also shows that the energy generated by HEG is fed back to power the LED, enabling energy recycling.

# References

S1 Huang, Z. *et al.* Flexible Moisture-Driven Electricity Generators Based on Heterogeneous Gels and Carbon Nanotubes. *ACS Applied Materials & Interfaces* **17**, 7916-7928, doi:10.1021/acsami.4c21266 (2025).

S2 Zhou, J., Ren, Z., Cui, X., Liu, X. & Lu, X. Bioinspired Interfacial Design of Robust Aramid Nanofiber Composite Films for High-Performance Moisture-Electric Generators. **n/a**, 2404840, doi:https://doi.org/10.1002/aenm.202404840.

S3 Cheng, Y. *et al.* Hydrogel-Based Moisture Electric Generator with High Output Performance Induced by Proton Hopping. **n/a**, 2500186, doi:https://doi.org/10.1002/adfm.202500186.

S4 Liang, J. *et al.* Directional Oxygen Defect Engineering in Black Phosphorus Aerogel for Flexible and Stable Moisture-Electric Generators. **n/a**, 2418834, doi:https://doi.org/10.1002/adfm.202418834.

S5 Gogoi, R. *et al.* Application of tandem heterojunction of metal oxides and hydroxides as robust and repairable moisture-electric generator. *Nano Energy* **131**, 110187, doi:https://doi.org/10.1016/j.nanoen.2024.110187 (2024).

S6 Zhan, L. *et al.* Moisture-triggered hybrid soft actuator and electric generator for self-sensing wearables and adaptive human-environment interaction. *Nano Energy* **132**, 110410, doi:https://doi.org/10.1016/j.nanoen.2024.110410 (2024).

S7 Irshad, M. S. *et al.* Controlled Self-Assembly and Photo-Thermal Activation of Viologen-Based 2D Semiconductors for Dual-Function Energy Management in All-Weather Applications. **n/a**, 2415101, doi:https://doi.org/10.1002/advs.202415101.

S8 Pan, X., Wang, Q., Jin, L., Ni, Y. & Rosei, F. Integrated paper-hydrogel structure for spontaneous and ultra-durable eco-friendly electricity generation. *Nano Energy* **136**, 110730, doi:https://doi.org/10.1016/j.nanoen.2025.110730 (2025).

S9 Hu, G. *et al.* All-In-One Carbon Foam Evaporators for Efficient Co-Generation of Freshwater and Electricity. **n/a**, 2423781, doi:https://doi.org/10.1002/adfm.202423781.

S10 Lin, J. *et al.* All Wood-Based Water Evaporation-Induced Electricity Generator. **34**, 2314231, doi:https://doi.org/10.1002/adfm.202314231 (2024).

S11 Mo, J. *et al.* Sulfated cellulose nanofibrils-based hydrogel moist-electric generator for energy harvesting. *Chem. Eng. J.* **491**, 152055, doi:https://doi.org/10.1016/j.cej.2024.152055 (2024).

S12 Liu, Q. *et al.* A Continuous Gradient Chemical Reduction Strategy of Graphene Oxide for Highly Efficient Evaporation-Driven Electricity Generation. **7**, 2300304, doi:https://doi.org/10.1002/smtd.202300304 (2023).
